# Photogated humidity-driven motility

**DOI:** 10.1038/ncomms8429

**Published:** 2015-06-11

**Authors:** Lidong Zhang, Haoran Liang, Jolly Jacob, Panče Naumov

**Affiliations:** 1 New York University Abu Dhabi, PO Box 129188, Abu Dhabi, United Arab Emirates; 2 Abu Dhabi University, PO Box 59911, Abu Dhabi, United Arab Emirates

## Abstract

Hygroinduced motion is a fundamental process of energy conversion that is essential for applications that require contactless actuation in response to the day–night rhythm of atmospheric humidity. Here we demonstrate that mechanical bistability caused by rapid and anisotropic adsorption and desorption of water vapour by a flexible dynamic element that harnesses the chemical potential across very small humidity gradients for perpetual motion can be effectively modulated with light. A mechanically robust material capable of rapid exchange of water with the surroundings is prepared that undergoes swift locomotion in effect to periodic shape reconfiguration with turnover frequency of <150 min^−1^. The element can lift objects ∼85 times heavier and can transport cargos ∼20 times heavier than itself. Having an azobenzene-containing conjugate as a photoactive dopant, this entirely humidity-driven self-actuation can be controlled remotely with ultraviolet light, thus setting a platform for next-generation smart biomimetic hybrids.

## Abstract (web summary)

Hygroinduced motion is fundamental for applications that require actuation in response to changes in atmospheric humidity. Here, the authors report an actuating material that is capable of fast and perpetual motion driven by humidity gradient incorporating a photoactive dopant that can be controlled by light.

## Graphical abstract

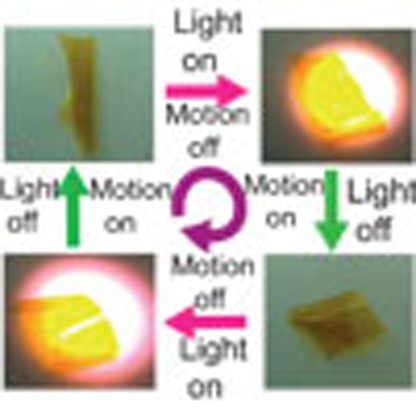

Smart actuating polymers are capable of mechanical reconfiguration by translating the energy contained within a chemical or physical stimulus into macroscopic changes of their shape and/or size^1^. In contrast to actuation driven by heat^2^, light^3^,^4^,^5^,^6^,^7^ and electricity^8^,^9^,^10^,^11^,^12^,^13^, soft actuators that respond to humidity are rather uncommon^14^,^15^,^16^,^17^,^18^. These dynamic elements represent artificial mimics of some biogenic actuating systems that can effectively utilize the day-and-night rhythm in the relative aerial humidity for passive burial or dispersion in arid conditions^19^. Nevertheless, the inherently slow kinematics, long response times and difficulties with external control of actuation caused by slow kinetics of water diffusion pose formidable practical challenges with their real-world applications as humidity-driven actuators. Being capable for utility of energy of spatial and/or temporal gradients in the chemical potential of water vapour, the biomimetic humidity-responsive materials are of particular interest to special applications that require contactless operation in the dark^15^,^16^,^17^,^20^.

We report herein an alternative approach to external control over humidity-induced actuation where a new actuating material that is capable of fast and perpetual motion driven by humidity gradient incorporates a photoactive dopant that can be controlled by light. When exposed to moisture on one face, the material utilizes the chemical potential of even very small humidity gradients for rapid and non-uniform exchange of water with the surroundings to propel itself through a series of swift and regular mechanical deformations. We demonstrate that excitation with weak ultraviolet light leads to contraction of the photoactive component and affects the motion kinetics, thereby providing means for dual-mode actuation and rapid gating of the humidity-driven motility by light.

## Results

### Preparation of the hybrid material

**Figure 1:**
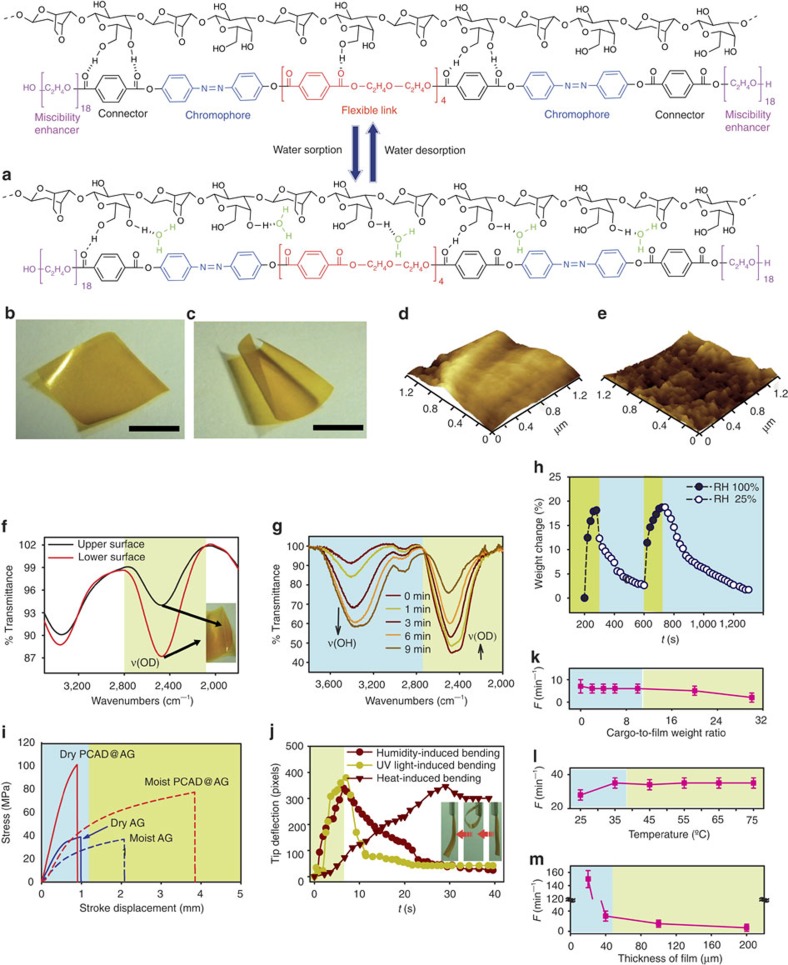
Structure and properties of a PCAD@AG actuator. (**a**) Chemical structure of the composite film and mechanism of water exchange. (**b**,**c**) Appearance of the hybrid film before (**b**) and after exposure to humidity from the surface (**c**). The length of the scale bar, 2 cm. (**d**,**e**) AFM images of the surface topography of dry film (**d**) and changes that occur on exposure of the film to humid air (**e**). (**f**) ATR−infrared spectra showing that the contact (lower) surface of a PCAD@AG film placed on a D_2_O-wetted substrate (filter paper) undergoes faster adsorption of D_2_O relative to the non-contact surface. (**g**) Time-dependent ATR–infrared spectra of film saturated by dipping in D_2_O for 10 s and kept in air (the excess D_2_O was wiped out from the surface). The spectra show rapid release of D_2_O and concomitant adsorption of H_2_O from the surroundings. (**h**) Variation of the weight of the PCAD@AG film with relative humidity of the air. Note that the film was not saturated with water in this experiment. (**i**) Typical stress–strain curves of films of PCAD@AG and non-doped AG before and after adsorption of water. (**j**) Typical patterns of deflection of the tip of a PCAD@AG film exposed to humidity, ultraviolet light and heat. The inset shows snapshots of the reversible humidity-driven bending. Note that the heat-induced bending is irreversible. (**k**–**m**) Effects of load weight, temperature and thickness on the turnover frequency (*F*, number of flips over time) of PCAD@AG film. The s.d. from three measurements are shown as error bars. AFM, atomic force microscopy; ATR, attenuated total reflection.

Agarose (AG) was selected as elastic hydrogel matrix. While AG has an extraordinary capacity for water adsorption with swelling capacity of ∼93% (ref. 20), the low melting point renders this material mechanically inapt for application as films are soft and collapse without external support. To attain concomitant mechanical robustness and photochemical response, a multifunctional photoresponsive poly(ethylene glycol) (PEG)-conjugated azobenzene derivative (PCAD) was prepared, characterized and embedded in the AG matrix (Fig. 1a; for details of the synthetic protocol see Supplementary Methods and Supplementary Fig. 1)^21^,^22^. As shown in Fig. 1a, the structure of the active component PCAD was designed to incorporate azobenzene units as chromophores/mechanophores for light harvesting and actuation, diethylene glycol as flexible component that provides elasticity, terephthalates as connecting functional groups that are also capable of hydrogen bonding, and PEG units for enhanced miscibility with the host matrix. The robust azobenzene unit was selected as photoactive component based on its well-known capability to effectively actuate polymers by *trans*–*cis* isomerization. For efficient energy harvesting and transduction to the matrix, two azobenzene units were incorporated into the backbone of PCAD. This number of chromophores was optimal, since incorporation of a single azobenzene unit afforded a semi-solid product and soft hybrid material with pronounced propensity for adhesion to the surface of the base, which strongly alleviated its performance in response to ultraviolet light. Higher number of azobenzene units, on the other hand, resulted in poor solubility and posed difficulties with preparation of the hybrid. Using terephthalate units as connectors, the two azobenzene units were coupled with each other through a tetra(terephthaloyl-diethylene glycol) segment as flexible linker. The flexibility and the short length of the central diethylene glycol unit were essential to provide conditions for simultaneous isomerization of both azobenzene chromophores without imposing mutual steric hindrance or conjugation-related constraints while retaining sufficient overall rigidity for actuation. The terephthalate connectors were also expected to facilitate the interaction with the host by providing additional sites for hydrogen bonding. Using another pair of terephthalates as connectors, the long and flexible PEG tails were introduced at both ends for improved miscibility, processability and elasticity of the hybrid film.

Films of the composite material (PCAD@AG) of variable thicknesses containing 10% (w/w) PCAD were casted onto glass substrates from a heated gelatinous mixture of AG and PCAD in dimethylformamide, and strips of varying shape were cut. To optimize the ratio between the guest and the host for best performance, films with three weight ratios of PCAD were prepared: 1, 10 and 15%. With 1% PCAD, corresponding to only 0.1% azobenzene units, the film responded weakly to ultraviolet irradiation (see below). The photoresponse from the film containing 15% PCAD was improved; however, the mechanical response to humidity was significantly reduced, possibly due to reduced gel content. The hybrid film containing 10% PCAD, equivalent to 1% azobenzene units, displayed optimal response to both light and humidity. PCAD@AG has superior mechanical strength relative to AG in both anhydrous and hydrated state (Fig. 1b,c). Unlike pure AG, which is gelatinous, soft and readily adheres to surfaces, free-standing films of PCAD@AG have cellophane-like appearance and are mechanically self-sustainable without mechanical support.

### Response to humidity

When a hanging PCAD@AG film is exposed to humidity from one side, it rapidly adsorbs water and swells, whereupon it quickly bends upwards (see the inset in Fig. 1j; Supplementary Movie 1). The deformation is directional and requires a gradient in humidity (see Supplementary Note 1). The film always bends or curls away from the source of humidity due to expansion on the exposed side caused by water sorption (Supplementary Fig. 2). When placed on a moist filter paper, the film rapidly curls and uncurls in succession and in opposite directions, thereby moving swiftly across the paper (Supplementary Movie 1). When removed from the filter paper, the motion of the film ceases instantaneously, but can be reacquired if the film is placed back onto the paper (Supplementary Movie 1). The motility can be turned on and off an indefinite number of times without apparent fatigue. The fatigue during the humidity-driven actuation of the film was studied with a special device where a film of PCAD@AG was exposed to humidity at a frequency of 20 min^–1^. The film was exposed during 9 min and the number of bending cycles during 1-min intervals was counted (Supplementary Movie 2). As can be inspected from Supplementary Fig. 3, the number of bending cycles of the film exhibits a periodic trend.

The material is very sensitive and responds even to very small gradients of humidity. When fixed to a support, thin strips deflect reversibly when approached with a finger and retract after the finger is removed (Supplementary Movie 3). When they are placed on the palm, the strips acquire traction instantaneously and move rapidly due to adsorption of moisture from the skin. We note that this ceaseless motion utilizes humidity gradient as the sole source of energy. The high sensitivity of the hybrid material to even very small humidity gradients is attributed to alteration of the secondary and tertiary structure of AG by PCAD. Pure AG is a known natural hydrogel that is capable of adsorption of considerable amount of water. However, the initial rate of adsorption is much higher than that of desorption. Consequently, the equilibrium between the adsorption and desorption is established slowly, which reflects in sluggish response to humidity gradients. When combined with PCAD, the rate of water adsorption slightly decreased, while the rate of desorption increased, and the equilibrium was established rapidly. This property is critical to the fast locomotion of PCAD@AG.

The capacity of PCAD@AG for water adsorption determined with a moisture balance at 80 °C on a film that was saturated with water in hot humid air for 2 h was 59.6% (Supplementary Fig. 2). Under identical conditions, a dry film lost only 2.8%. In a series of (re)hydration experiments, we concluded that the optimal water content for rapid locomotion is 20−40%; lower water content is insufficient for efficient swelling, while at higher water content the film motility is alleviated by enhanced adhesion to the substrate surface. To demonstrate the capability to perform mechanical work, a 200-μm thick film was loaded with a small metal rod as cargo and placed on a moist paper (Supplementary Movie 4). Relative to the unloaded film the loading did not have any detectable effect on the rolling frequency at cargo-to-film weight ratio of up to about 20:1 (Fig. 1k). To determine the change of volume during swelling, a rectangular film (4.66 × 2.5 × 0.40 mm^3^) was placed on a moist filter paper for 30 min. The volume of the film expanded from 4.66 to 5.27 mm^3^, giving a volume expansion of 13% (Supplementary Fig. 4).

### Mechanism of water adsorption

The progress of water adsorption and desorption was monitored with attenuated total reflection−infrared spectroscopy after the film was brought in contact (10 s) with D_2_O-pre-wetted filter paper. The evolution of the band at ∼2,500 cm^−1^ indicated faster adsorption of D_2_O on the lower face of the film (the surface that was in contact with the substrate) relative to the upper face (Fig. 1f). This non-uniform adsorption of water is responsible for anisotropic swelling that drives the deformation. Atomic force microscopy images of the surface showed that the adsorption of water alters the surface morphology by formation of ridges and increased roughness from 0.52 to 1.33 nm (Fig. 1d,e). The high surface-area-to-volume ratio facilitates the water desorption, until an equilibrium between the water in the loaded film and in the surroundings is established. This equilibrium is dynamic; it can be shifted and re-established on a minute time scale simply by variation of relative humidity, and is reflected in weight of the film (Fig. 1h; note that to study the reversibility, the film was not saturated with water). The desorption was monitored *in situ*, after soaking of the film in D_2_O for 10 s, removal of the excess heavy water on the surface and exposure of the D_2_O-saturated surface to air. As the film exchanged water with the surroundings, the ν(OD) band at ∼2,500 cm^−1^ rapidly decreased with concomitant increase of the ν(OH) band at ∼3,400 cm^−1^ (Fig. 1g). Changes in relative humidity can perturb this equilibrium and have a strong effect on the mechanical properties of the film. Dry PCAD@AG film is elastic and has a Young's modulus (*E*=4.71 GPa) almost double that of pure dry AG (*E*=2.32 GPa; Fig. 1i). After adsorption of water, the critical elongation (elongation at point of rupture) increased from 2.91 to 11.37% (for comparison, for pure AG film, the value increased from 3.16 to only 6.25%). As it can be concluded from the related changes in the infrared spectra (Supplementary Fig. 5), this change in mechanical properties is due to diffusing water molecules, which compete with PCAD for hydrogen bonding to AG (Fig. 1a; Supplementary Note 2 and Supplementary Fig. 10).

### Humidity-driven mechanical work

**Figure 2:**
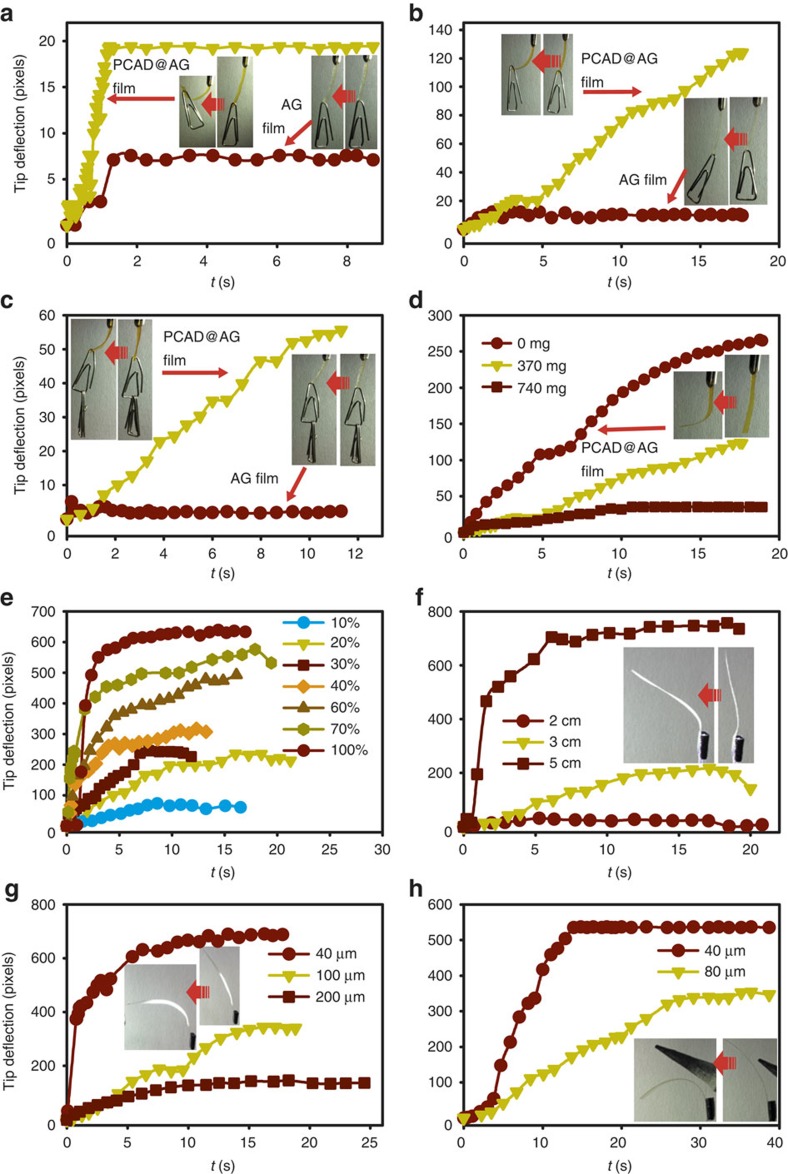
Performance of bending PCAD@AG films. The *y* axis (in pixels) is the relative deflection of the tip of the film (for details on the kinematic analysis, see Supplementary Methods). The inset in each figure shows snapshots of the bending films. (**a**) Humidity-induced bending of films of PCAD@AG (16 mg) and pure AG (15 mg) of size 2 cm × 0.5 cm × 100 μm loaded with a cargo of 370 mg. (**b**) Humidity-induced bending of films of PCAD@AG and pure AG of size: 2 cm × 0.5 cm × 200 μm carrying a cargo of 370 mg (note the different scale on the *y* axis with **a**). (**c**) Performance of doped versus undoped film in humidity-induced bending (film size: 1 cm × 0.5 cm × 200 μm, cargo weight: 1,110 mg). (**d**) Effect of cargo weight on humidity-induced bending (film size: 2 cm × 0.5 cm × 200 μm, cargo weight: 370 mg and 1,110 mg). (**e**) Effect of ultraviolet power on photochemical bending (film size: 4 cm × 0.5 cm × 40 μm, ultraviolet power: 2–20 mW cm^−2^). (**f**) Effect of the length of the strip on the photochemical bending (ultraviolet light power: 20 mW cm^−2^). The numbers refer to the length of the strips. (**g**) Effect of film thickness on photochemical bending (ultraviolet power: 20 mW cm^−2^). (**h**) Effect of film thickness on the thermally induced bending of the films. The tip of a heated metal pin used as heat source is shown in the inset.

Hanging films of PCAD@AG affixed at their upper terminus that respond to non-uniform exposure to humid air by reversible bending can operate as cantilever cranes, which harvest energy of the chemical potential contained within the humidity gradient to convert it to mechanical work (Supplementary Movie 5). Lifting tests showed that hybrid films of any size performed better relative to films of pure AG under identical conditions (Fig. 2a–c; for details of the kinematic analysis see the Supplementary Methods). A 16 mg, 100-μm-thick film of PCAD@AG was capable of hoisting a cargo of 370 mg to a height of 2 cm with largest tip deflection in 1.2 s (Fig. 2a; Supplementary Fig. 6). This actuation is four orders of magnitude faster than that reported for a pNIPAm-microgel-based device (pNIPAm stands for poly(*N*-isopropylacrylamide)^14^. The work output and power density of this actuator (4.53 J kg^–1^ and 3.78 W kg^–1^, respectively) are also superior to those of pure AG films (1.13 J kg^–1^ and 0.94 W kg^–1^). Thicker films (200 μm) did not perform better (2.80 J kg^–1^ and 0.16 W kg^–1^; Fig. 2b and Supplementary Fig. 6), however, shortening of the film enhanced the bending force. A cargo of 1,110 mg was lifted to a height of 0.6 cm with work output 5.01 J kg^–1^ and power density 0.44 W kg^–1^ (Fig. 2c; Supplementary Fig. 6). For pure AG film of the same size, the work output was only 0.012 J kg^–1^ and the power density was 0.001 W kg^–1^. A maximum load of 740 mg on the PCAD@AG film (Fig. 2d) induced much lower stress (∼0.007 MPa) than the maximum elastic stress for moist (∼35 MPa) and dry (∼100 MPa) film (Fig. 1i). Thus, the film always operates in its elastic regime and efficiently overcomes the increased shear and stress. We estimated the theoretical limit of maximum load of a 200-μm-thick film within the elastic regime to a remarkable ∼3.5 kg (for details of the calculation, see the Supplementary Methods), although this theoretical limit is probably practically inaccessible at a reasonable rate.

### Kinematic analysis of hygromechanical response

**Figure 3:**
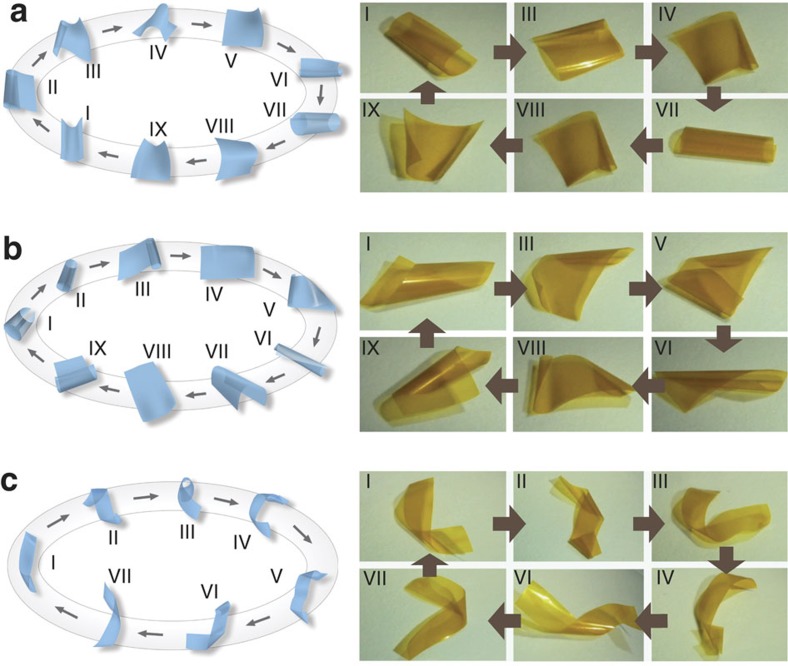
Mechanism of hygroinduced locomotion of PCAD@AG films. (**a**) Square-shaped film, (**b**) rectangular film and (**c**) film strip. For each aspect ratio and stage of locomotion, both cartoons and snapshots of the real film are shown.

The kinematics and mechanism of locomotion depend on the aspect ratio of the film. A film cut into a square undergoes a locomotive cycle composed of nine stages (Fig. 3a; Supplementary Movie 6). When the film is placed on a moist substrate, the expansion of the surface of the film facing the substrate due to adsorption of water exceeds that of the opposite (upper) surface, whereby the film curls up (I). As the gravity centre rises, the film becomes mechanically unstable and slumps to one side (II). The film then curls up from one corner and rolls over horizontally (III). The upper face of the film turns over the lower face and comes into contact with the moist substrate (IV). As the film unfolds, one corner starts rolling up again (V). The continual water adsorption and desorption eventually drives the film to curl up into a tubular shape (VI). Mechanical instability then causes the tube to roll and slide (VII). The upper face of the film slides down again (VIII), and due to asymmetric swelling, the unfolded film continues to curl up to start a new cycle (IX). A PCAD@AG film cut into a rectangular shape undergoes a series of sequential locomotive processes similar to those observed with a square-shaped film (Fig. 3b; Supplementary Movie 6). Briefly, the film curls up (I) to form a short tube (II) and topples over (III). It then unfolds, whereby the upper face turns down (IV). The film then rolls up from one corner (V) and shapes up into a long tube (VI), which unfolds (VII), cocks up from one corner (VIII) and rolls from one side to start a new cycle (IX).

Locomotion of a film cut into a strip proceeds by a different scenario (Fig. 3c; Supplementary Movie 6). Asymmetric water adsorption first causes the film to distort with one end curling up and the other end curling down (I). As the gravity centre is displaced, the film becomes mechanically unstable whereupon both curled termini twist (II). The water adsorption and desorption further cause both ends of the film to topple over and contact the moist substrate (III). The accumulation of strain causes sudden unfolding of the film at both ends (IV). The surface of the film tilts towards the moist substrate and adsorbs water faster than the opposite face, inducing curling to the opposite direction due to unbalanced swelling (V). The shift of the barycenter subsequently causes the film to twist into a spiral shape at both ends (VI). The film then rolls over and the twisted structure unfolds to start a new cycle (VII).

The thickness of the film and temperature affect its turnover frequency. For a strip of PCAD@AG with thickness of 20 μm the frequency was *F*≈150 min^−1^, which represents a fivefold improvement in performance relative to PEE−PPy films of similar thickness^16^. Increase of the thickness to 200 μm enhances the stiffness and decreases the rolling frequency to *F*≈5 min^−1^ (Fig. 1m). The temperature also affects the water exchange kinetics. At 25 °C, the kinetics of evaporation of water from the moist substrate is relatively slow which sets a small humidity gradient and decelerates the motion. Contrary to intuitive expectations, higher temperature (∼75 °C) does not increase rolling frequency because the greater water gradient at higher temperature is counterbalanced with faster water desorption from the surface of the film. At the optimal temperature for rolling ∼35 °C, the quasistationary state with balanced rates of water adsorption and desorption is rapidly established (Fig. 1l). We note that this temperature is close to physiological temperature, which opens prospects for utility of this material in biomechanical devices. As a rather rudimentary proof of the concept of utilizing the humidity gradient in biomechanics, we designed a miniature soft robot that is capable of continual motion and can also carry cargos when exposed to anisotropic source of humidity (Supplementary Movie 7). In another application, we fabricated a coiled strip of the film that can coil around an object with a prolate shape when exposed to humidity in a motion, which resembles the coiling of plant tendrils (Supplementary Movie 7).

### Response to ultraviolet light

The photoactive azobenzene groups were introduced to the hybrid material as PCAD to elicit response to light and to exert external control of motion. Indeed, when excited with heat-filtered continuous-wavelength ultraviolet light, the isomerization of the azobenzene units in PCAD induced macroscopic photomechanical bending of the film (Supplementary Movie 8). The bending was more pronounced at higher power densities (Fig. 2e)^23^,^24^. When irradiated with light of identical power (20 mW cm^−2^), thinner films (40 μm) were more elastic; they deflected faster and to a higher degree relative to thicker films (Fig. 2g). Having other parameters fixed, longer ribbons showed faster and more pronounced bending in response to ultraviolet light than short strips (Fig. 2f). However, unlike the humidity-induced bending, where the bending always occurred in a direction opposite to that of the flow of humidified air, the films exposed to ultraviolet light appeared to bend in random directions, in line with the different mechanism of mechanical deformation. The AG matrix has a relatively low melting point (Supplementary Fig. 7) and the motion PCAD@AG film could be, in principle, due to heating effects^25^,^26^. However, control experiments showed that heat-induced deformations are permanent and the film does not recover its original elasticity (Fig. 2h). Thus, heating effects can be ruled out as a main factor for the mechanical reconfiguration.

The *trans-to-cis* isomerization of the azobenzene units in PCAD@AG film was confirmed by the ultraviolet–visible (ultraviolet–vis) spectrum of a film that was irradiated with continuous-wavelength ultraviolet light (*λ*=325–385 nm) and monitored throughout the course of irradiation (Supplementary Fig. 8). The effect on the ultraviolet–vis spectrum of the chromophore in dimethylformamide solution was also recorded. The absorbance at *λ*=365 nm decreased gradually with exposure to ultraviolet irradiation, while the intensity of the bands at *λ*=296 and 479 nm increased, both observations being consistent with *trans*-to-*cis* isomerization of the azobenzene units. The isomerization took place within 35 s of ultraviolet irradiation, and the *trans/cis* ratio in the photostationary state was ∼1.5. To confirm the reversibility of the PCAD isomerization, a PCAD@AG film was irradiated with light (*λ*=325–385 nm) for 5 min, and then bleached with *λ*>400 nm. The bleaching restored the original spectrum (Supplementary Fig. 8); however, the reverse (*cis*-to-*trans*) isomerization was much slower compared with the forward (*trans*-to-*cis*) isomerization. These spectral changes and the azobenzene as the sole group that undergoes photoisomerization in PCAD were confirmed by comparison with a solution of pure PCAD in dimethylformamide. The spectral changes exhibit great similarity; however, in line with the less sterically constrained environment in solution, both reactions were faster in solution (*trans*–*cis* isomerization to a photostationary state required only 14 s, with ∼45% of the azobenzene units in the *cis* form).

### Kinematic analysis of photomechanical response

**Figure 4:**
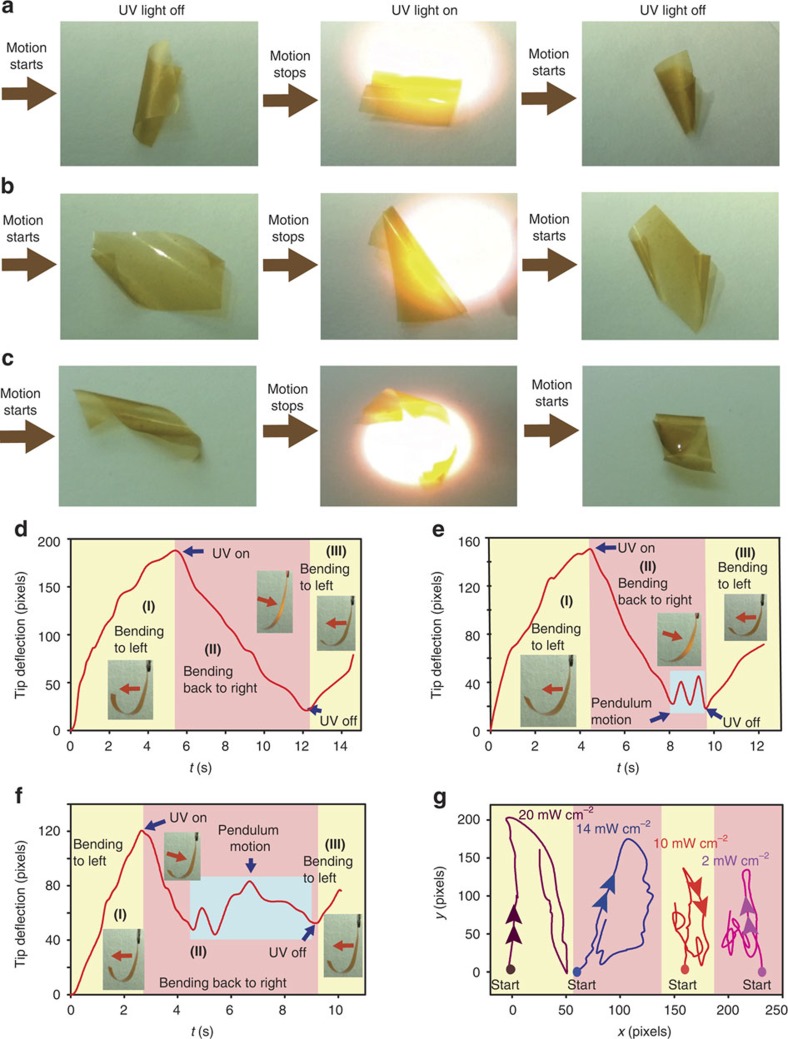
Photoinduced control and kinematic analysis of the hygroinduced locomotion of PCAD@AG films. (**a**–**c**) Effect of the aspect ratio: square-shaped film (**a**), rectangular film (**b**) and film strip (**c**). (**d**–**f**) Effect of ultraviolet excitation power: 20 mW cm^−2^ (**d**), 10 mW cm^−2^ (**e**), and 2 mW cm^−2^ (**f**). (**g**) Two-dimensional kinematic traces of the film tip.

As shown in Supplementary Movie 9, when a PCAD@AG film in motion was exposed to ultraviolet light with power of 20 mW cm^−2^, its motion ceased instantaneously (Fig. 4a−c; Supplementary Note 3 and Supplementary Fig. 11). The motility resumed as soon as the excitation was terminated. This switching on and off of the humidity-driven motility can be repeated many times, and is brought about by the isomerization of the azobenzene chromophores in PCAD (we confirmed that a film of pure AG moved very slowly over a moist surface and ultraviolet light did not have any effect on the motion; Supplementary Movie 10). This exercise indicates the possibility to remotely ‘gate' the humidity-driven motility with light. To demonstrate the macroscopic effects of photogating of humidity-driven actuation, a bending film was exposed to ultraviolet light (Fig. 4d–g; Supplementary Movie 9). Humid air was continually supplied laterally to the film to maintain relative humidity within the range of mechanical bistability. This induced slow deflection from the source of humidity due to expansion of the contact surface (stage I, Fig. 4d; for details of the kinematic analysis see Supplementary Methods). Exposure to ultraviolet light from the opposite direction resulted in expansion of the illuminated surface and suppression of the humidity-induced unidirectional bending. This action reverted the bending direction, causing the film to deflect away from the light source and against the air flow, that is, in the direction of increasing humidity gradient (stage II). After the ultraviolet light was switched off, the film switched the bending direction again and resumed its original unidirectional bending (stage III).

By modulating photoexcitation, the extent of deflection of the film can be finely tuned and the direction of bending can be inverted on a time scale of <1 s. The neat reverse bending that is maintained by a combination of steady flow of humid air and constant ultraviolet excitation power 14–20 mW cm^−2^ (Fig. 4d) can be modulated by using low excitation power. With weak excitation (10 mW cm^−2^, Fig. 4e), the counter-bending proceeds smoothly during 3.5 s up to the point where the opposing bending moments caused by light and humidity become balanced and the film enters a region of bistability (in respect to stimulation with humidity and light). The system then starts to bounce back and forth between regions of strong excitation/low humidity and weak excitation/high humidity. This appears as readily observable oscillatory motion of the tip of the film that can be sustained by maintaining strictly controlled conditions (see the highlighted portion of the kinematic plot in Fig. 4e). The latent period preceding the onset of this oscillatory motion depends on the excitation power. Decrease of the power to 2 mW cm^−2^ shortens the latent period to 1.9 s, but it also results in damped oscillations (Fig. 4f). Thus, not only the amplitude but also the trajectory and the oscillations of this bistable system can be controlled by using light (Fig. 4g).

## Discussion

**Figure 5:**
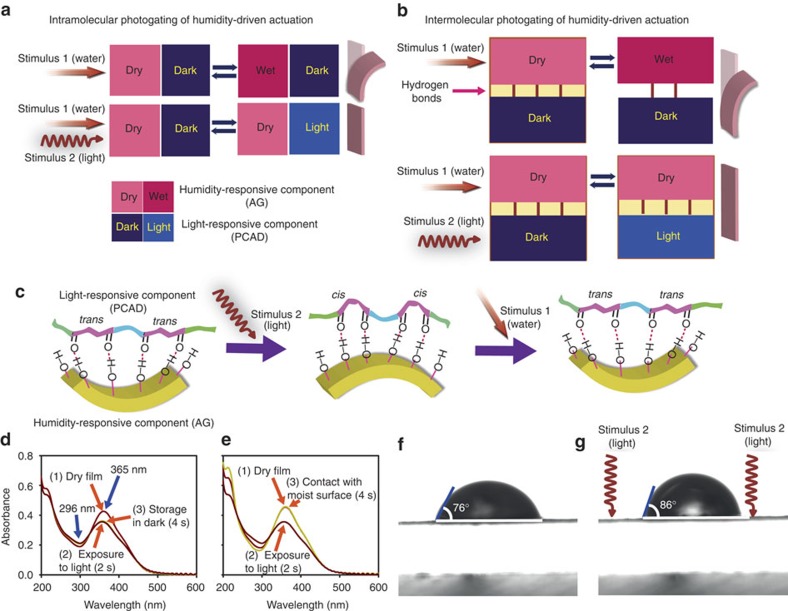
Mechanism of photogated humidity-driven actuation. (**a**) With intramolecular photogating of humidity-driven actuation, the units that respond to humidity and light are part of the same molecule. While action of stimulus 1 (exposure to humidity) alone induces bending, action of stimulus 2 (exposure to light) prevents the bending driven by stimulus 1. (**b**) With intermolecular photogating, such as that in PCAD@AG, the units that respond to humidity and light are different molecules coupled by intermolecular interactions. Action of stimulus 1 (humidity) induces bending. Action of stimulus 2 (light) prevents the bending by isomerization and decreases the affinity of AG for adsorption of water. Note that stimulus 1 also affects the hydrogen bonding network between PCAD and AG. (**c**) Mechanism of photogating of humidity-induced motility. The *trans* form of azobenzene groups in a PCAD@AG film bent by humidity is converted to the *cis* form. Exposure to humidity facilitates the isomerization of the *cis* to the *trans* form and prevents bending. (**d**,**e**) Photoinduced actuation gated by humidity monitored by ultraviolet–vis spectroscopy. In **d**, the sample was first exposed to ultraviolet light for 2 s whereby the *trans* form (the characteristic strong peak in the visible region) was partially converted to the *cis* form that was thermally stable in the dark. As shown in **e**, on exposure to humidity, the *cis* form was reverted completely to the *trans* form in <4 s, and thus the humidity counterbalanced the isomerization of the chromophore. (**f**,**g**) Evidence of the humidity-induced actuation gated by light. In **f**, a water droplet sits on the PCAD@AG film at a contact angle of 76°. As shown in **g**, irradiation with ultraviolet light increased the contact angle to 86° and enhanced the hydrophobicity of the surface, thus alleviating the propensity of the material for adsorption of water.

The mechanism of control over the motility of PCAD@AG with light raises a question regarding the underlying mechanism, where the hindrance of motion can occur at a molecular level, as a result of entanglement of the molecular changes induced by the two stimuli, or at a macroscopic level, as a result of the action of two independent but opposing forces. In a conventional intramolecularly gated system, such as are some thermally controlled photochromic molecules, the gating is accomplished by exerting control over the effect of one of the stimuli (light) by action of the other (heat), both stimuli operating on sensing units that are located on the same molecule (for a schematic of a hypothetical intramolecularly photogated hygroresponsive system, see Fig. 5a). The switching induced by the gated stimulus can be directly controlled by activation of the gating stimulus. PCAD@AG is a hybrid material and can be considered an intermolecularly gated system, in the sense that the two sensing units are represented by two chemically distinct albeit coupled (through intermolecular interactions) molecules. Namely, each of the two components (PCAD and AG) is a sensing unit that responds to different stimulus. The two units are connected and communicate with each other through an extensive hydrogen bonding network (Fig. 5b). In effect, changes induced by the effect of external stimulus on one of the components affect the other component indirectly, through the hydrogen bonded linkages. Consequently, exposure of the hygroresponsive component (AG) to humidity can switch that component between two states (‘dry' and ‘wet'). Application of light to the photoresponsive component (PCAD), on the other hand, switches that component from ‘dark' to ‘light' state and prevents the first component (AG) from switching between the ‘dry' and ‘wet' states, thereby effectively gating its response. An additional complication in this two-component system comes from the fact that not only does water adsorption affect the hygroresponsive component (AG) by direct hydration, but it does so by partial disruption of the hydrogen bonding linkages between AG and PCAD (Fig. 5b).

The hypothesis of gating of the response in PCAD@AG at a molecular level was tested by studying the effects of light and humidity on the same surface of the film (note that being a two-component hybrid, the outcome of the combined effects of humidity and ultraviolet light will depend on whether they impinge on the same surface; neighbouring PCAD and AG molecules will be simultaneously affected only when the two stimuli are applied on the same face of the film or when the film is very thin, as is the case with all films studied here). First, the intermolecular photoinduced gating of the response of the film to humidity was examined by measuring the contact angle between the film surface and a water droplet as indicator of the hydrophilicity, and thus of the affinity of the film for water adsorption. A drop of water placed on the film stood at an average contact angle of 76° (Fig. 5f). Only when ultraviolet light was shined on the same surface, the average contact angle increased to 86° without observable changes in surface roughness, showing that the surface had become more hydrophobic. Supplementary Fig. 9 shows similar measurements performed on pure PCAD. We conclude that isomerization of the azobenzene chromophore to the *cis* form decreases the propensity of the material to adsorb water. This change is reversible, in support of the hypothesis that the response to humidity can be gated by light (Fig. 5c). To test whether the photoresponse can be gated by the action of humidity, the visible absorption of the *trans*-azobenzene group was used as a direct measure of the concentration of the *trans* isomer in the film. To ascertain thermal stability, PCAD in AG was first partially converted from *trans*-to-*cis* form by exposure of the PCAD@AG film to ultraviolet light, and stored in the dark. The *cis* form did not isomerize to any detectable extent to the *trans* form in 4 s (Fig. 5d), confirming that the *cis* form was thermally stable in the dark. In a second experiment, after the *cis* form was generated by excitation with ultraviolet light, the film was brought in contact with a moist paper. The ultraviolet–vis spectrum showed that exposure to humidity accelerated the decay of the *cis* from, and the *trans* form was recovered completely in <4 s (Fig. 5e). It should be noted that humidity does not affect the spectrum of pure PCAD (see Supplementary Note 4 and Supplementary Fig. 15). This result provides evidence that the exposure of the film to humidity facilitates the *cis*-to-*trans* isomerization, in support of the gating model. The fast *cis*-to-*trans* isomerization accounts for the fast recovery of the humidity-driven motion once the excitation has been terminated (Fig. 4). If taken together, these results confirm that the mechanisms of photocontrol of humidity-induced bending and hygrocontrol of photoinduced bending of PCAD@AG are true molecular-level gating processes.

**Figure 6:**
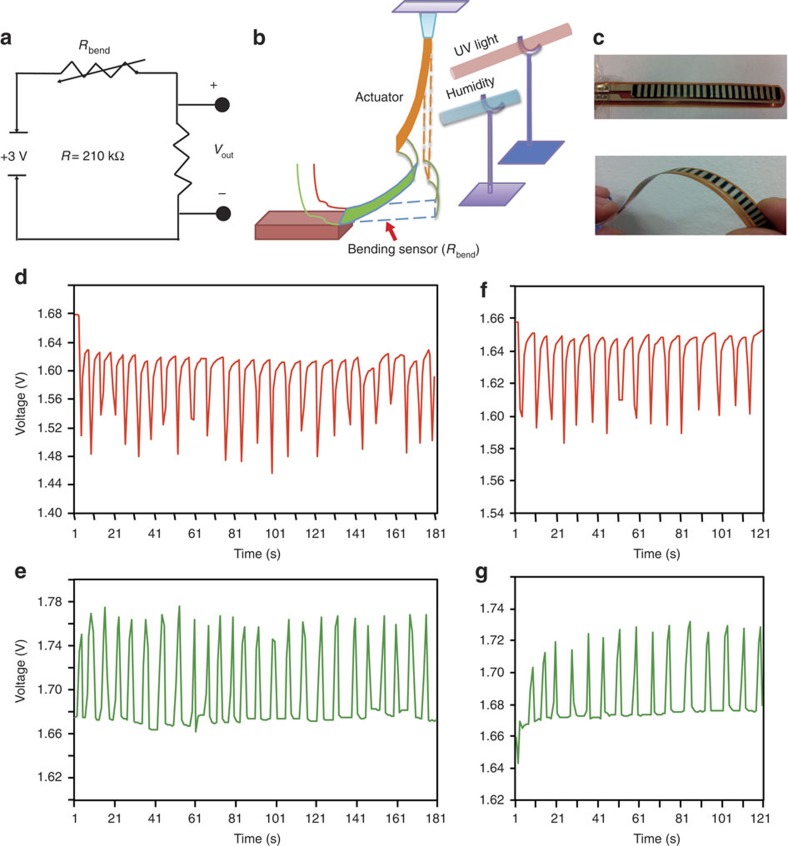
Voltage divider controlled by humidity- and light-driven PCAD@AG actuator. (**a**) Schematic of the voltage divider circuit. (**b**) A sketch showing the principles of operation of the bending sensor. When humidity or light is applied on PCAD@AG film, it bends to trigger bending of the sensor, whereupon the resistance changes affect the distribution of the voltage in the circuit. (**c**) Without applying a bending moment, the sensor used in the circuit is originally straight, but can be bent to change the resistance. (**d**,**e**) When the PCAD@AG actuator is driven by humidity, the output voltage oscillates with the bending of the sensor in the high-resistance regime (**d**) and in the low-resistance regime (**e**). (**f**,**g**) As the actuator is irradiated with light, the output voltage regularly alter with the sensor bending in the high- (f) and low- (g) resistance regime. The voltage output of the circuit in **a** is given by *V*_out_=*R*_bend_*V*/(*R*+*R*_bend_), where *R*=210 kΩ and *V*=3 V.

As a proof-of-concept application to energy conversion by the hybrid film, a voltage divider was designed and attached to a humidity/light-responsive sensor composed of the PCAD@AG composite as active component coupled to a flex sensor (*R*_bend_ in Fig. 6a). Bending of the PCAD@AG film by exposure to humidity or ultraviolet light induces bending of the sensor (Fig. 6b). The change of resistance of the sensor affects the after-voltage of the electrical circuit. By humidity-induced regular and reversible motion of the actuator, the voltage spectrum of the bending sensor could be varied in a wide sensing range (176–215 kΩ) when operating in the high-resistance regime and narrower sensing range (145–167 kΩ) in the low-resistance regime. Within the 176–215 kΩ range the output voltage was 1.63–1.48 V (Fig. 6d), while within the range 145–167 kΩ the output voltage was 1.77–1.67 V (Fig. 6e). When light was used to drive the actuator, the bending sensor showed similar efficiency to that observed with humidity-driven processes with wider sensing range (172–186 kΩ) on the high-resistance side that corresponds to output voltage range of 1.65–1.48 V (Fig. 6f). When bending on the low-resistance side (154–167 kΩ), the output voltage was 1.73–1.67 V (Fig. 6g). The output voltage was recorded in 1 s intervals. Apparently, humidity-driven bending of PCAD@AG film was able to direct the voltage divider within wider range relative to light-induced process. This design allows for a remote adjustment of the output voltage of the circuit simply by changes in environmental humidity or by exposure to ultraviolet light, which could have potentials for remote control of electrical circuits in hazardous environments. In a more advanced setup, the sensing unit coated with the humidity-active material that is exposed to humidity would normally be physically separated from the electronics so the water would not affect the long-term operation of the device.

In addition to the higher turnover frequency, relative to the humidity-responsive material PEE-PPy (pentaerythritol ethoxylate-polypyrrole) that was recently reported by Langer *et al*.,^16^ the material described here presents several advantages while also providing new insights into the mechanism of humidity-driven locomotion. Films prepared from PCAD@AG are highly optically transparent, insulating, and can be prepared by a simple mixing-and-casting procedure, thus providing opportunity for control of performance by varying the composition. The use of AG as a readily available, cheap and ostensibly biocompatible natural hydrogel opens prospects for applications in bioengineering. As a more important conceptual difference, unlike PEE-PPy where the response to humidity occurs due to hydration and reversible hydrolysis and esterification of the borate ester bonds in the polyol borate network, the actuation of PCAD@AG is driven by rearrangement of the hydrogen bonding network in a series of dehydration–rehydration cycles. Being devoid of hydrolysis, this rearrangement is related to lower activation energies, proceeds rapidly and its kinetics is limited only by the diffusion rate. The different mechanism of actuation is the most viable reason for the fast response to humidity gradients observed with PCAD@AG.

In summary, we combined the strong hygroscopic capability of a natural hydrogel (AG) with photoactivity of a flexible synthetic photoactive polymer based on azobenzene-containing photoactive poly(ethylene glycol) conjugate in a smart hybrid material that is capable of self-actuation in response to gradient in humidity and of photomechanical response by excitation with ultraviolet light. The resulting hybrid material can harness the chemical potential of humidity gradient and convert it to mechanical work. By rapid and non-uniform swelling, the water adsorption elicits remarkable shape reconfiguration including reversible bending, helical twisting and continual rolling or flipping. When exposed to humidity, slender films of the material can perpetually curl and uncurl to advance rapidly over a moist surface and transport cargos of weights that are nearly 20-fold their own weight. When anchored, actuating elements fabricated from this material can bend and swiftly lift objects 85 times their own weight. Furthermore, the presence of photoactive azobenzene groups enables gating of this remarkable motility with ultraviolet light. Even though light-triggered humidity-induced swelling of hydrogels with modified surface topographies is known^27^,^28^,^29^, light-controlled humidity-driven motion of a free-standing film of hydrogel has not been reported before this work. The actuating material reported here can operate in the dark and could be utilized to convert humidity gradients into electrical power in low-power generators that can be fuelled by humidity and/or light. The advantage of the material with dual responsive described is that it can operate both in the dark, in a humidity-driven mode of operation, and under ultraviolet light, in light-driven mode of operation. This could be useful for continual switching and control of devices installed in remote and hardly accessible areas that can operate using sunlight during daytime and using changes in humidity during night.

## Methods

### Preparation and characterization of the PCAD@AG composite film

PCAD was prepared in a series of consecutive condensations (for details on the synthetic protocol, see the Supplementary Methods and Supplementary Figs 12, 13 and 14). To 10 ml of dimethylformamide, 1.0 g of AG was added with vigorous stirring to obtain a homogenous mixture. The mixture was heated at 110 °C, until the AG powder was dissolved completely and 0.1 g PCAD in dimethylformamide was injected into the solution. The stirring was terminated when the formation of bubbles ceased and casted onto pre-treated microscopic slides. Films were obtained after drying in air over 2 days. Films with different thickness were prepared using glass moulds. The water content of the PCAD@AG film before and after exposure to humidity was determined with moisture analyser (Mettler Toledo MJ33) at 80 °C. Dry PCAD@AG film was kept in vacuum overnight at 50 °C for equilibration (final sample weight: 1.171 g). To load a sample with water, the film was exposed to water vapour during 2 h (water temperature: 60 °C; final sample weight: 4.295 g).

### Infrared spectroscopy

An Agilent Cary 630 FTIR spectrometer was used to detect the water adsorption and desorption of the films at 25 °C. D_2_O was used instead of water to shift the absorption bands to a spectral region that is devoid of absorption by atmospheric humidity. After soaking in D_2_O for 10 s, the excess D_2_O from the surface of the film was removed carefully with a dry filter paper. The time-dependent infrared spectra were immediately recorded (attenuated total reflection mode) from a fixed area of the film (0.5 × 0.5 cm).

### Ultraviolet–vis spectroscopy

The ultraviolet–vis spectra were recorded in transmission mode on a Shimadzu UV–3600 UV–Vis–NIR spectrometer at 25 °C. The films were attached to a quartz plate. To study the *trans*-to-*cis* isomerization of the azobenzene units in PCAD@AG, a film of pure AG was employed for background check and also as reference in the sample measurements. The spectrum of the PCAD@AG film was recorded before irradiation (*t*=0 s). The PCAD@AG film was irradiated with ultraviolet light from a medium-pressure Hg lamp (*λ*=325–385 nm) and the spectral changes were recorded with time until subsequent spectra were indistinguishable. To study the *cis*-to-*trans* isomerization of the azobenzene units, the PCAD@AG film was first irradiated with ultraviolet light at *λ*=325–385 nm for 5 min and the spectrum was recorded (*t*=0 s). The film was then exposed to light from the ultraviolet lamp filtered with a sharp-cut filter (*λ*>400 nm) and the spectral changes were monitored with time. The solution-state spectrum was recorded from a solution of PCAD in dimethylformamide in a 1-cm quartz cuvette against pure solvent as background.

### Surface imaging

Model 5500 atomic force microscope from Agilent was used to inspect the surface topology of the films.

### Thermal analysis

The thermal properties were analysed with differential scanning calorimeter Q2000 (TA) equipped with an auto sampler at a heating rate of 10 °C min^–1^ in the temperature range 25–200 °C. Tzero aluminum pans were used for the measurement. The thermogravimetric analysis was performed with SDT Q600 instrument (TA) at a heating rate of 10 °C min^–1^ using aluminum pans. The temperature range was 20–800 °C and dry N_2_ gas was used as carrier gas.

### High-resolution mass spectrometry

The spectra were recorded on electrospray quadrupole/time-of-flight-type mass spectrometer MicrOTOF-Q (Bruker Daltonics, Germany) operated in positive ion mode. The performance and resolution were verified using Tunemix (Agilent Technologies, USA) with resolution at *m/z* 1,222 of 12,000. Mass calibration was achieved using Tunemix (internal calibration) between *m/z* 50 and 3,000 in the same acquisition mode that affords accuracy of 10 p.p.m. One acquisition consisted of infusing the sample at 50 μM in CH_3_CN:H_2_O (1:1) during 2 min, followed by 1-min infusion of Tunemix. The capillary voltage was set to 4,500 V and the ion energy at 5 eV. The system was controlled by Compass Hystar (Bruker Daltonics, Germany). Before each sample injection, a blank consisting of CH_3_CN:H_2_O (1:1) was injected. Data analysis was performed with the software Data Analysis 4.

### Tensile testing

All specimens were tested at 25 °C. In the tensile tests, a sample of rectangular shape (4 × 0.5 cm) was clamped by the grips of movable and stationary fixtures in a screw-driven device using universal testing machine equipment AG-50KNXu (Shimadzu, Japan) that pulls the sample until breaking while recording the applied load and elongation. The gage length was fixed to 30 mm and test speed was 18 mm min^–1^, which corresponds to strain rate of 0.01 s^–1^. The load cell and extensometer were calibrated before use. The tests complied with rules specified by the international standard norms. The average value and s.d. were calculated from a minimum of three samples.

### Contact angle measurements

The contact angle measurement of the control and irradiated PCAD@AG films was carried out by the sessile drop method using an OCA 15EC contact angle analyser (Dataphysics instruments GmbH, Germany). Each value of the contact angle was taken as an average value measured from five different samples of the hybrid film fabricated under the same experimental conditions. To determine the contact angle of pure PCAD, PCAD (50 mg) was dissolved in 2 ml dimethylformamide at 50 °C, and the solution was cast onto a pre-cleared glass slide. The compact layer obtained after the solvent was completely evaporated at 50 °C in a vacuum oven was used in the contact angle measurements.

### Measurement of the volume change on water adsorption

A rectangular PCAD@AG film (4.66 × 2.50 × 0.40 mm) was placed on a moist paper (water content ∼50% by weight). The environment temperature was 25 °C and the relative humidity was 28–29%. The size of the film was measured in 5-min intervals. The total time for exposure was 30 min.

### Study of the reversibility of film bending induced by humidity

A film of PCAD@AG affixed to a base was placed over the mouth of a bottle filled with a lukewarm water (40 °C). The film and the mouth of the bottle were separated by a sheet of paper that could be shifted back and forth by a mechanical rotor at a frequency 20 min^–1^. The film was exposed to humidity in regular 1-min intervals during 9 min and the number of cycles during each interval was counted.

## Additional information

**How to cite this article**: Zhang, L. *et al*. Photogated humidity-driven motility. *Nat. Commun.* 6:7429 doi: 10.1038/ncomms8429 (2015).

## Supplementary Material

Supplementary Figures, Supplementary Notes, Supplementary Methods and Supplementary References. Supplementary Figures 1-15, Supplementary Notes 1-4, Supplementary Methods and Supplementary References.

Supplementary Movie 1 Bending of strip of PCAD@AG exposed to humidity from the right side. (2) Motion of a film of PCAD@AG over a moist paper. (3) Motion of a film of PCAD@AG over a moist paper and cessation of motion when the film is removed from the moist paper.

Supplementary Movie 2 A film of PCAD@AG responds when approached with a finger due to the humidity gradient created by the skin moisture. (2) Motion of the film placed on the palm.

Supplementary Movie 3 Motion over moist paper of a film of PCAD@AG loaded with cargo. Motility at different cargo-to-film weight ratio is shown.

Supplementary Movie 4 Motion of a film of PCAD@AG exposed to heat-filtered UV light from a medium-pressure Hg UV lamp.

Supplementary Movie 5 A film of PCAD@AG operating as a cantilever crane. The weight of the film here is 13 mg, and that of the cargo is 1110 mg.

Supplementary Movie 6 Kinematic analysis of motion of films of PCAD@AG with aspect ratios 1 : 1 (square), 1 : 2 (rectangle), and 1 : 5 (strip).

Supplementary Movie 7 Two examples of potential use of the humidity-responsive polymer. (1) This soft robot carrying the logo of New York University Abu Dhabi crawls by converting the chemical potential of the humidity gradient into mechanical work. (2) A strip of PCAD@AG simulates a plant tendril by wrapping itself around a solid support (glass rod) when exposed to humidity.

Supplementary Movie 8 Humidity-driven motion of films of PCAD@AG modulated by UV light.

Supplementary Movie 9 UV light does not affect motion of a film of pure agarose over moist paper. (2) UV light does not affect motion of a film of pure agarose over moist paper. (3) Heat does not affect motion of a film of PCAD@AG over a moist paper. (4) The motion of PCAD@AG over a moist paper can be controlled by UV light.

Supplementary Movie 10 Reversibility of motion of PCAD@AG film by repeated short exposures to water vapor.
