# Supplementary material for: Photogated humidity-driven motility
Source: Nat Commun. 2015 Jun 11;6:7429. doi: 10.1038/ncomms8429 (PMC4490392; doi:10.1038/ncomms8429)
Supplement: Supplementary Figures, Supplementary Notes, Supplementary Methods and Supplementary References. — Supplementary Figures 1-15, Supplementary Notes 1-4, Supplementary Methods and Supplementary References. [file ncomms8429-s1.pdf]

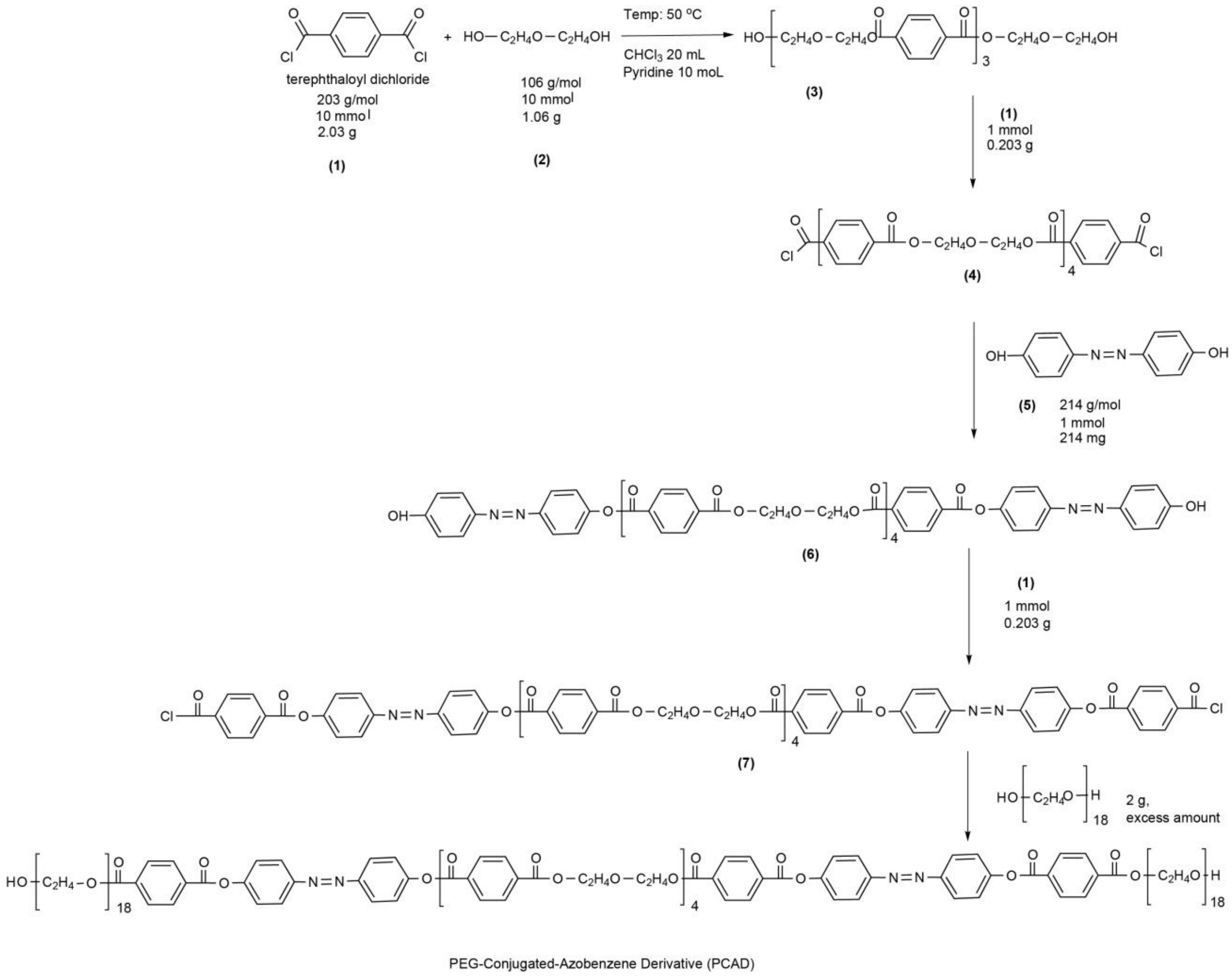

Supplementary Figure 1. Synthetic sequence for synthesis of PCAD.

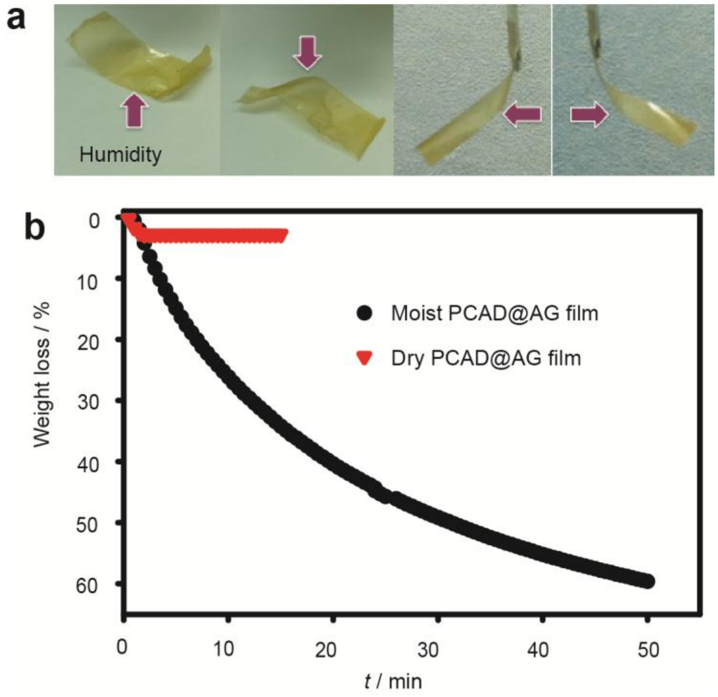

Supplementary Figure 2. Directional bending of the PCAD@AG film induced by humidity, and dehydration of dry and loaded PCAD@AG monitored with a moisture balance. (a) When the lower surface of the film is brought in contact with the moist filter paper, the film bends up (top left panel). When the upper surface is exposed to humid air, the film bends to the lower side (top right panel). Similarly, a freely hanging film always bends away from the source of humidity (bottom panels). The arrows show the direction from where humid air is supplied. (b) The large weight loss (59.6%) of loaded PCAD@AG film at 80 °C indicates strong water sorption ability of the film. In this experiment, the film was saturated with water by exposure to hot water vapor (~70 ^{o}C) for 2 hours before the weight loss was recorded.

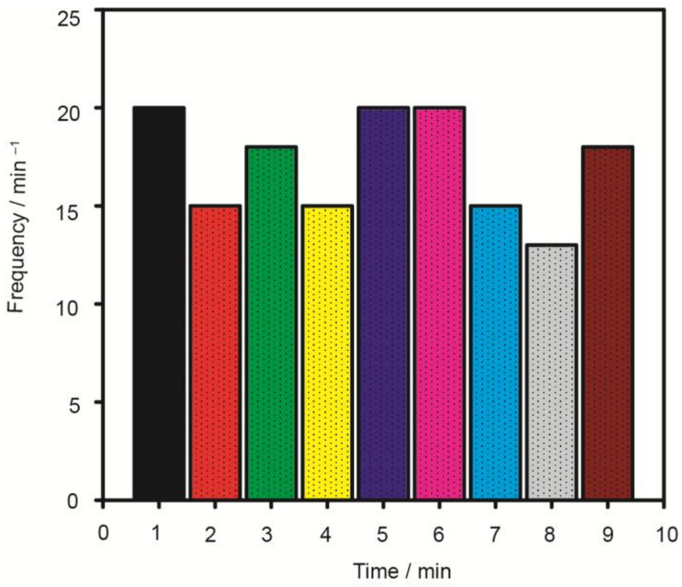

Supplementary Figure 3. Bending cycle performance of PCAD@AG film in response to repetitive exposure to humidity. To study the reversibility of the actuation of the film, a simple device was constructed. A film of PCAD@AG was affixed over the mouth of a bottle that was filled with warm water (40 ^{o}C). The film and the mouth of the bottle were separated by a sheet of paper that could be shifted back and forth by a mechanical rotor at a frequency 20 minute^{-1}. The film was exposed to humidity in regular 1-minute intervals during 9 minutes and the number of bending cycles during each interval was counted. During the 1st minute, the bending of the film kept the pace with the exposure to humidity (20 minute^{-1}; Supplementary Movie 2 shows the motion of the film during the 1st minute). During the following 3 minutes, the bending frequency became slightly lower than the exposure, presumably due to the continual and fast bending motion during the 1st minute, which increased the overall softness of the film. The reduced bending frequency brought to temporary recovery of the stiffness, and the bending frequency rose again to 20 minute^{-1} during the 5th and 6th minute. This was followed by another decrease in frequency during the 7th and 8th minute and increase (to 18 minute^{-1}) during the 9th minute. Thus, the bending cycles of the film exhibited a periodic trend, however with fairly good uniformity over time.

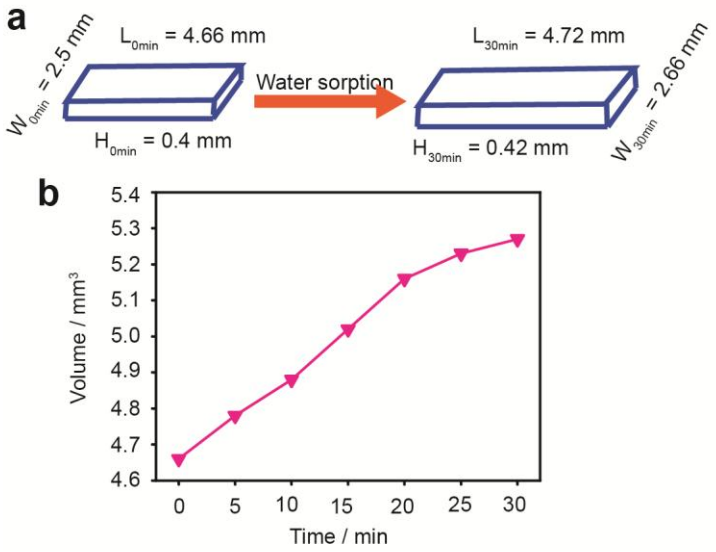

Supplementary Figure 4. Volume change of PCAD@AG film by exposure to a wet surface. (a) Sketch showing the expansion of a rectangular PCAD@AG film with time during 30 minutes after it was placed on a moist filter paper and changes in absolute dimensions. (b) The temperature of the environment was 25 ^{o}C and the relative humidity of the air in the room was 28–29%. By exposure for 30 min, the film expanded from 4.66 mm^3 to 5.27 mm^3, giving a volume expansion of 13%.

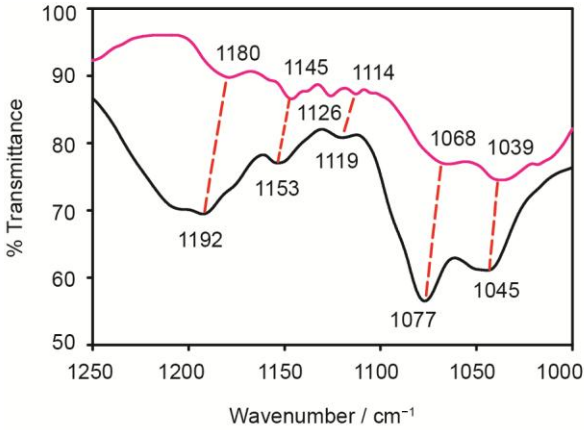

Supplementary Figure 5. ATR-IR spectra of the PCAD@AG film before (pink line) and after (black line) D_2O treatment. The blueshifts in the range 1000–1250 cm^{-1} in effect of the D_2O treatment could indicate the hydrogen bonds between the carbonyl groups of PCAD and the hydroxyl groups of AG are disrupted due to water sorption.

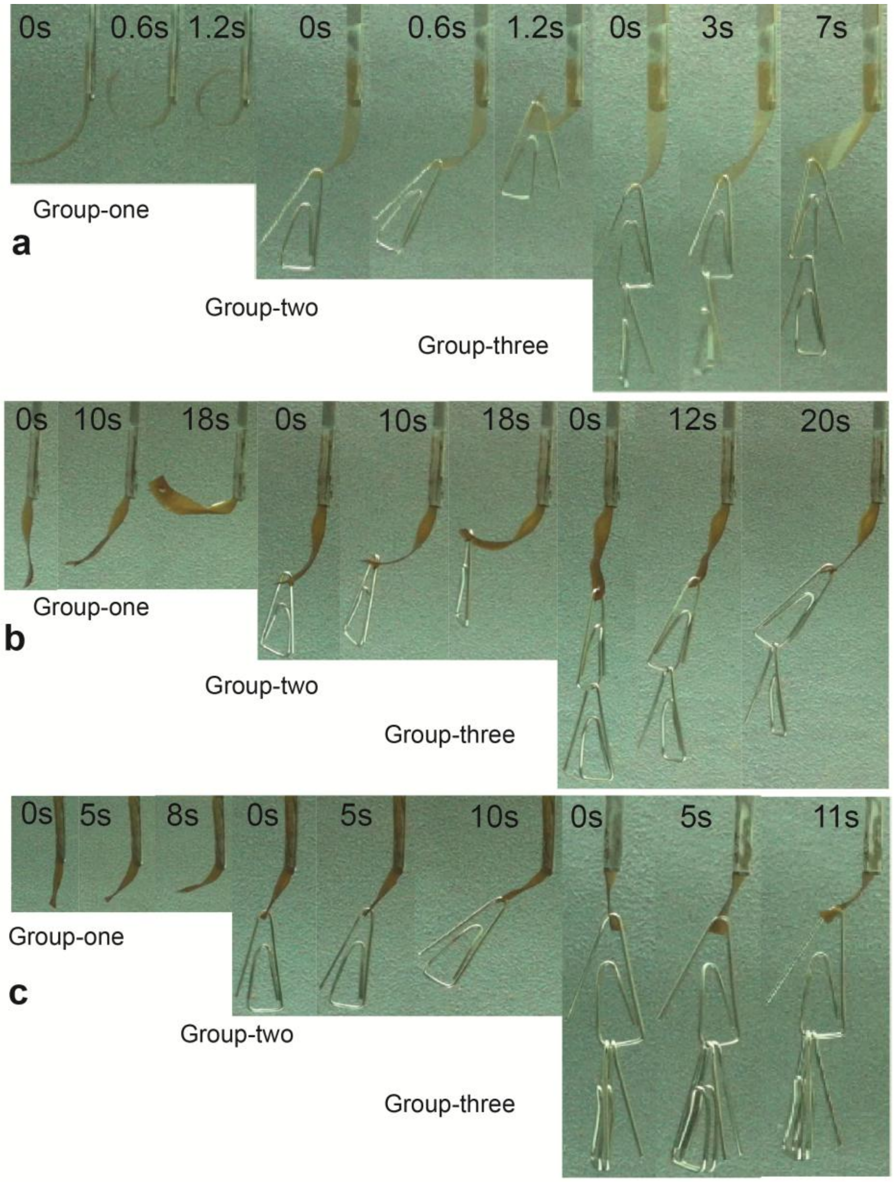

Supplementary Figure 6. Bending motion of PCAD@AG film with cargo. (a) Effect of cargo weight on bending of 2-cm, 100-μm-thick PCAD@AG film. Film dimensions: 2 cm (L) × 0.5 cm (W) × 100 μm (H); film weight: 16 mg; temperature of humid air: ~35 ^{o}C; cargo weight: 0 mg ("Group-one"), 370 mg ("Group-two"), 740 mg ("Group-three"). (b) Effect of cargo weight on bending of 2-cm, 200 μm-thick PCAD@AG film. Film dimensions: 2 cm (L) × 0.5 cm (W) × 200 μm (H); film weight: 26 mg; moisture temperature: ~35 ^{o}C; cargo weight: 0 mg ("Group-one"), 370 mg ("Group-two"), 740 mg ("Group-three"). (c) Effect of cargo weight on bending of 1-cm, 200 μm-thick PCAD@AG film. Film dimensions: 1 cm (L) × 0.5 cm (W) × 200 μm (H); weight: 13 mg; moisture temperature: ~35 ^{o}C; cargo weight: 0 mg ("Group-one"), 370 mg ("Group-two"), 1110 mg ("Group-three").

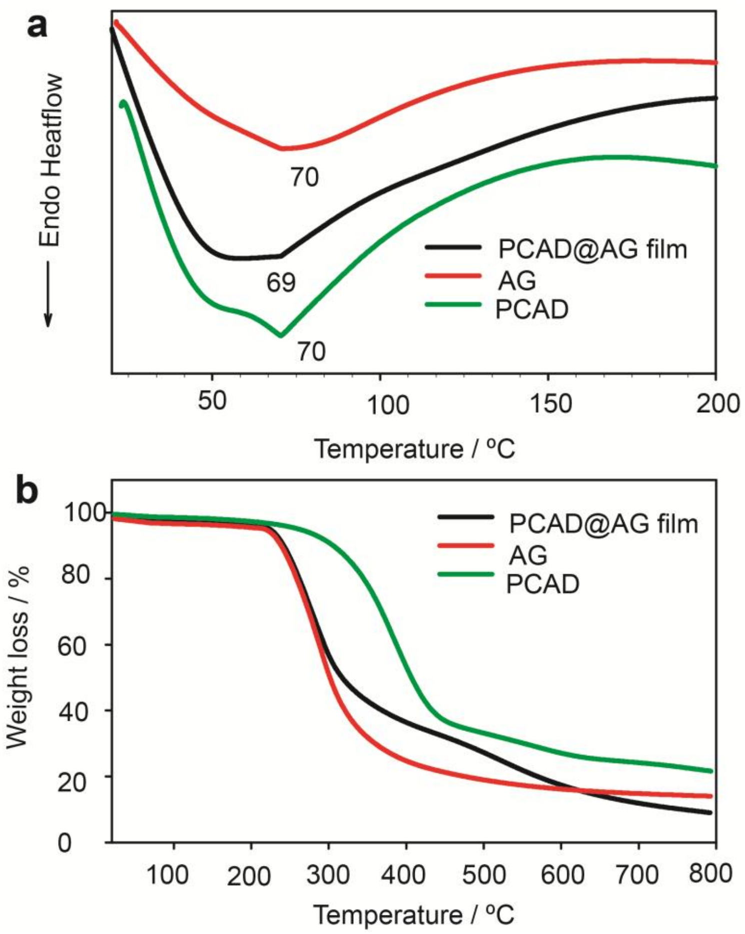

Supplementary Figure 7. Thermal analysis of PCAD, AG and the PCAD@AG hybrid film. (a) Differential scanning calorimetry (the numbers are the temperatures of the peaks in ^{o}C). (b) Thermogravimetric analysis.

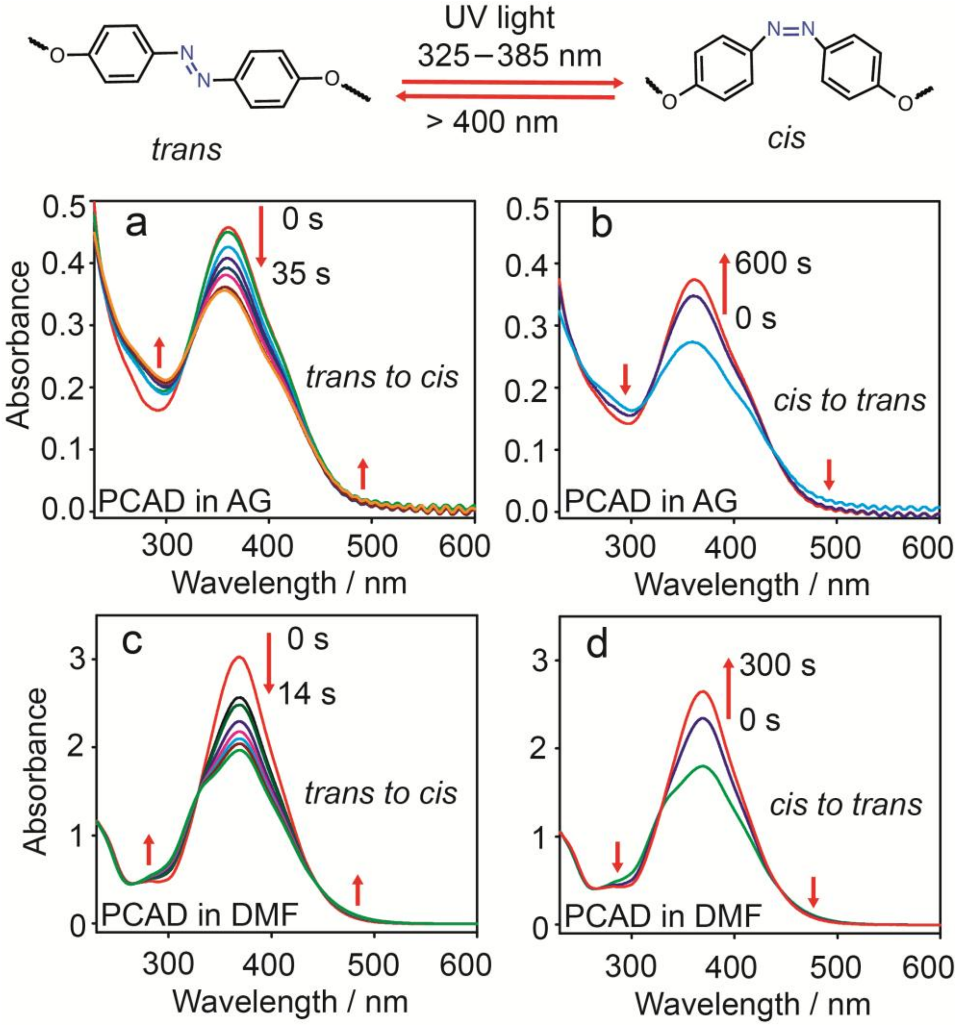

Supplementary Figure 8. Time-dependent UV-vis spectra of irradiated PCAD in AG and in dimethylformamide (DMF) solution. (a and b) UV-vis spectra of freshly prepared PCAD@AG film recorded with pure AG film as reference; in (a) the film was irradiated with UV light with λ = 325–385 nm from a medium-pressure Hg lamp, while in (b) the film was first irradiated by UV light at λ = 325–385 nm for 5 minutes and subsequently exposed to light with λ > 400 nm. (c and d) UV-vis spectra of PCAD in DMF; in (c) the solution was irradiated with UV light at λ = 325–385 nm, while in (d) the PCAD solution was first irradiated with UV light at λ = 325–385 nm for 5 minutes and then exposed to light with λ > 400 nm. The wavelengths λ > 400 nm were selected by using a sharp-cut filter to remove the high-energy part of the spectrum.

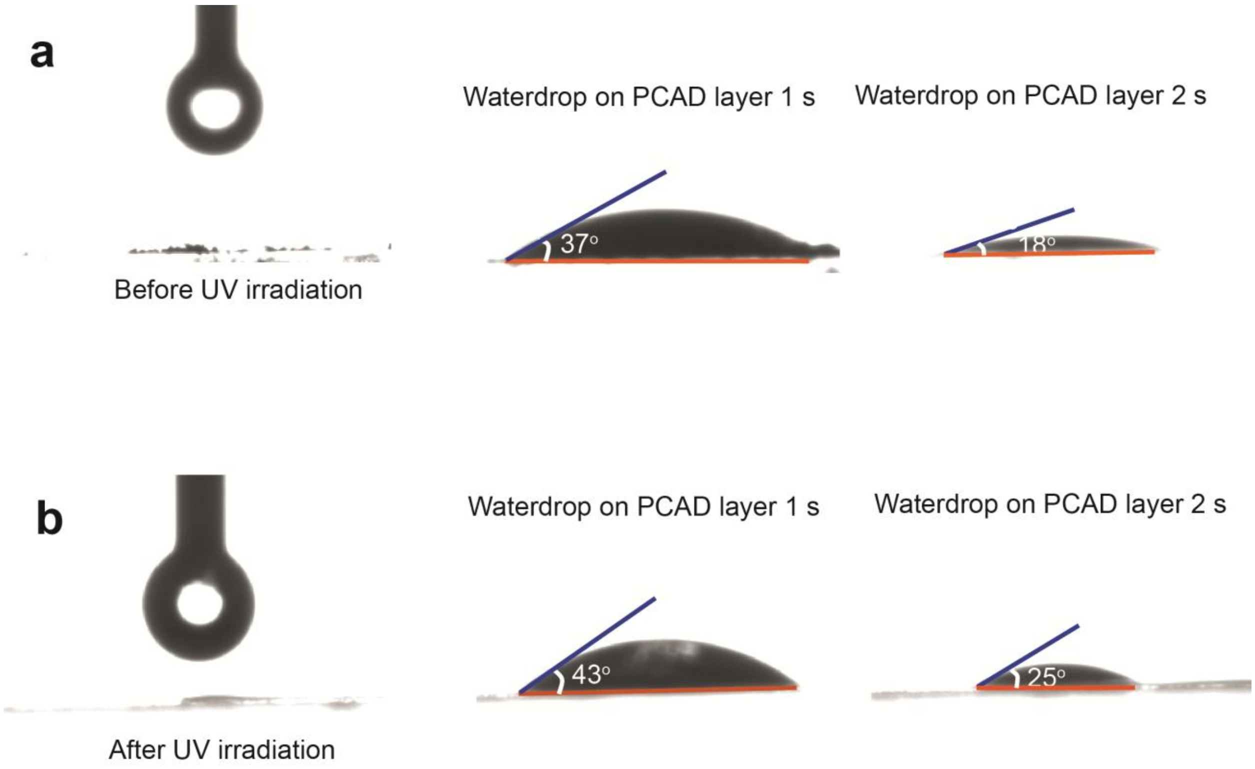

Supplementary Figure 9. Light-induced change of wettability of pure PCAD. (a) Before UV irradiation, and (b) after UV irradiation. The results show that wettability of PCAD decreased slightly by irradiation of UV light, in agreement with the response from PCAD@AG film (see Figure 5 f and g in the main text).

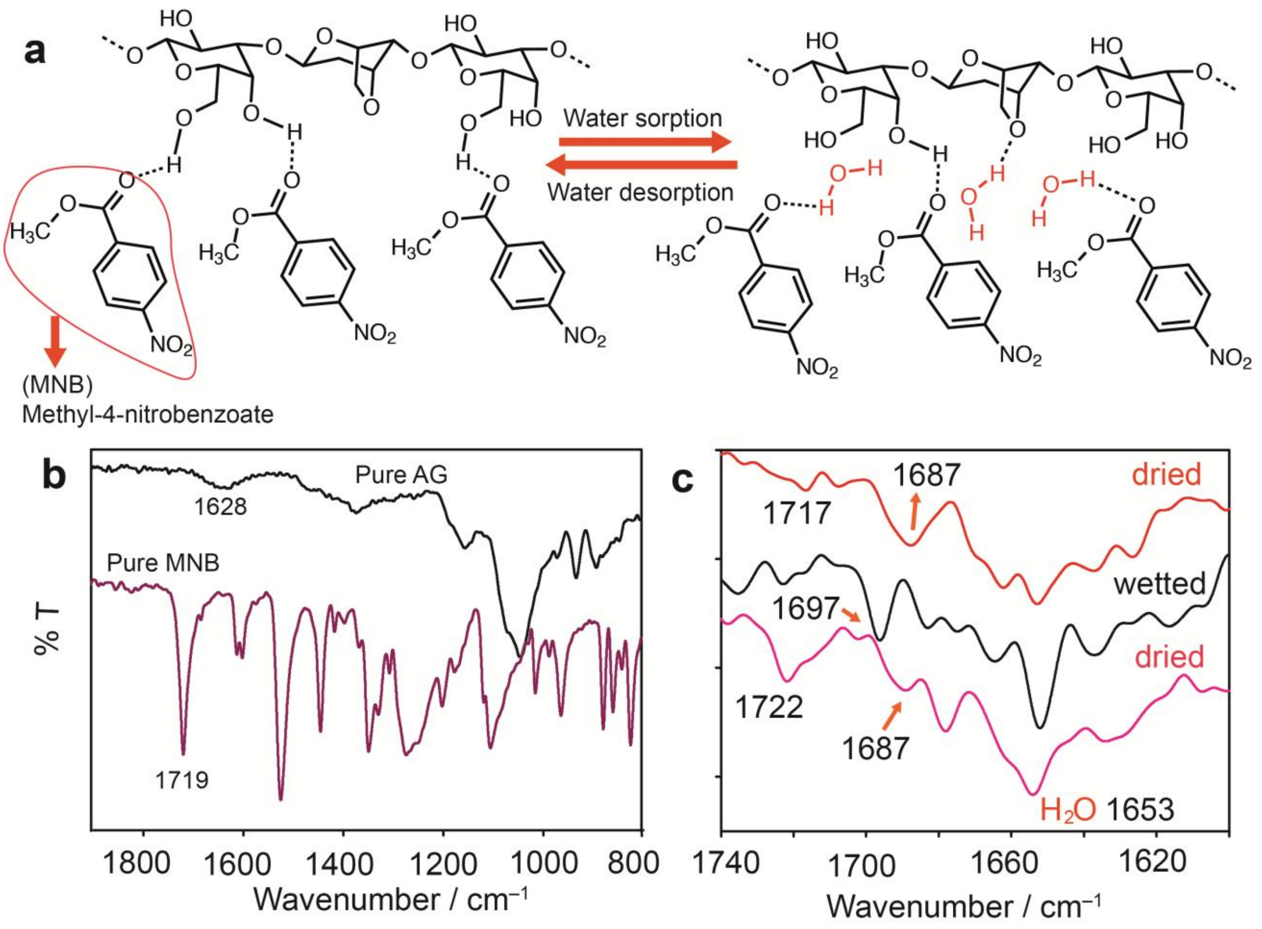

Supplementary Figure 10. IR spectra of a model for PCAD@AG where PCAD was replaced with methyl-4-nitrobenzoate (MNB). (a) Approximate model of the changes in hydrogen bonding network in MNB@AG that occur upon adsorption and desorption of water. (b) Infrared spectra of pure AG and pure MNB for comparison. (c) IR spectra of MNB@AG before and after hydration, and after drying.

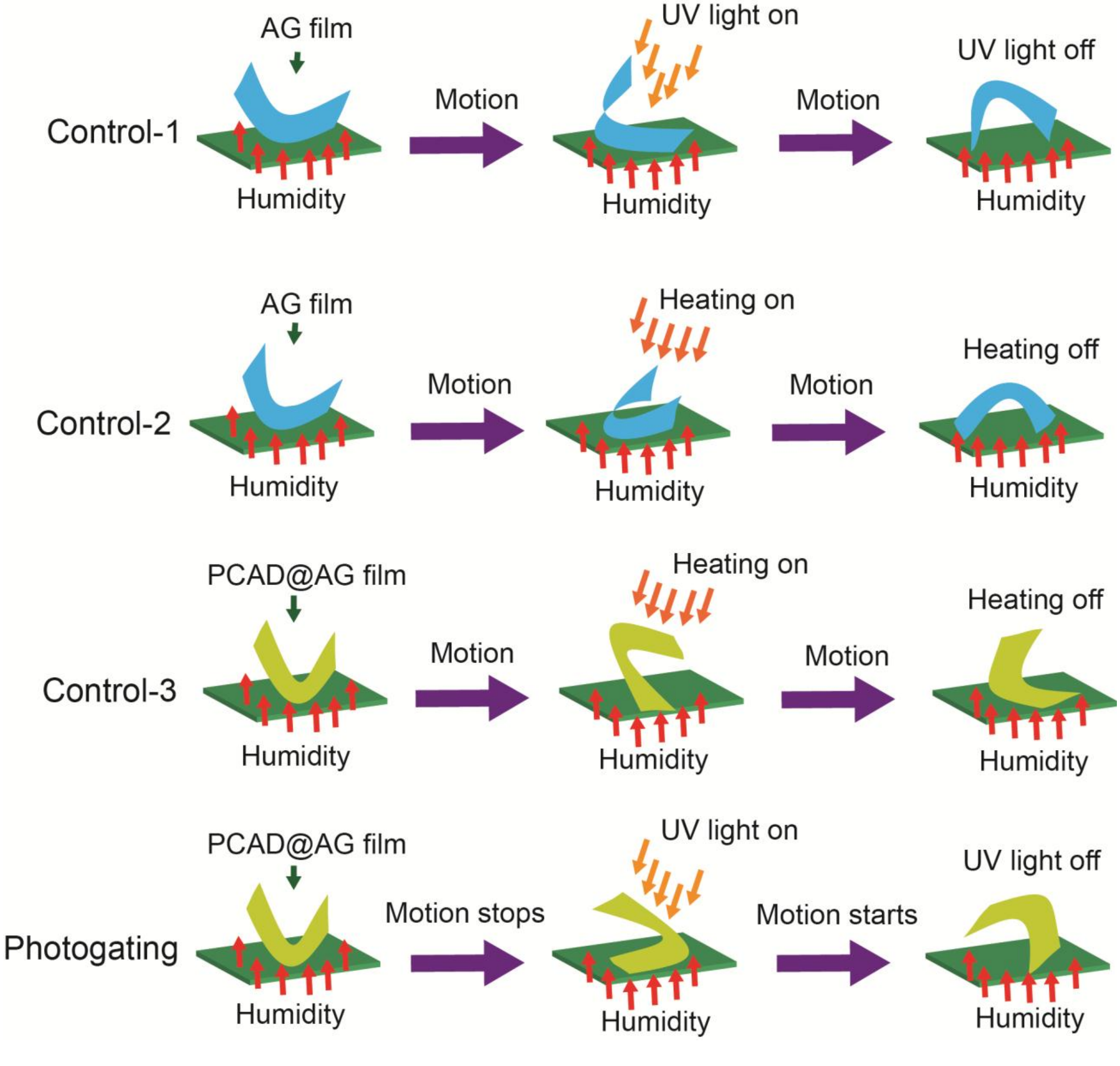

Supplementary Figure 11. Schematic of the experiments that show the effects of humidity and light on films of pure agarose (AG) and PCAD@AG.

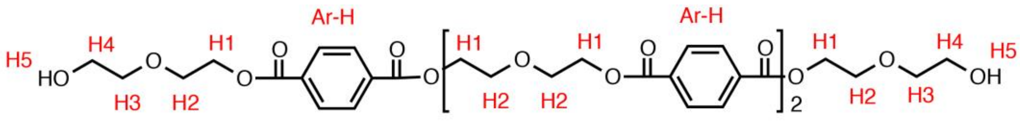

(3)

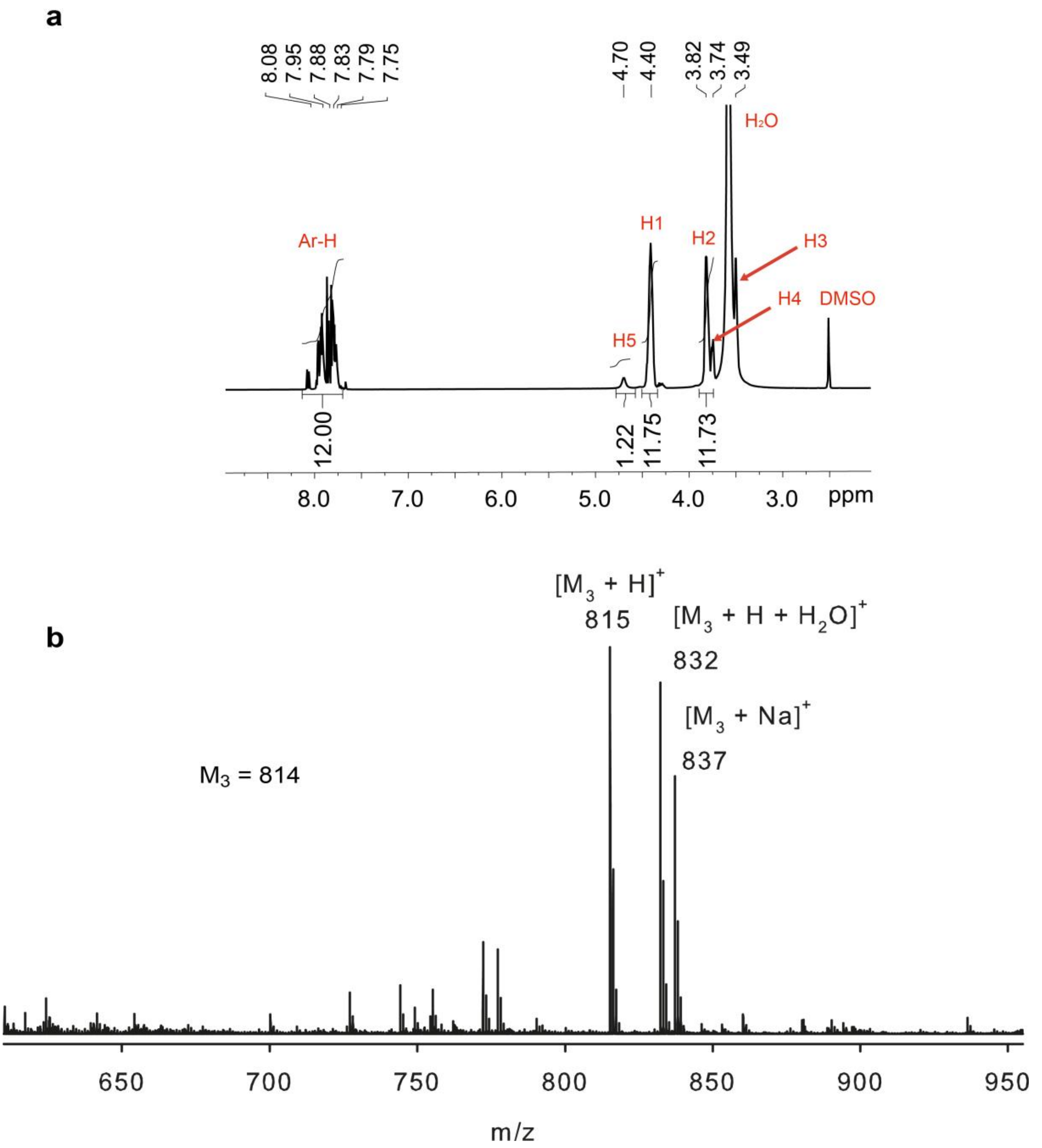

Supplementary Figure 12. Structure and characterization of product 3. ^{1}H NMR spectrum (600 MHz, DMSO-d_6): δ 3.49 (s, 4H, –CH_2–), 3.74 (s, 4H, –CH_2–), 3.82 (s, 12H, –CH_2–), 4.40 (s, 12H, –CH_2–), 4.70 (s, 2H, –OH), 7.75–8.08 (m, 12H, Ar–H); ESI–MS (*m*/*z*): $[M]^+$ calcd. for M_3 ($C_{40}H_{46}O_{16}$), 814; found, 815 $[M_3+H]^+$; 832 $[M_3+H+H_2O]^+$; 837 $[M_3+Na]^+$.

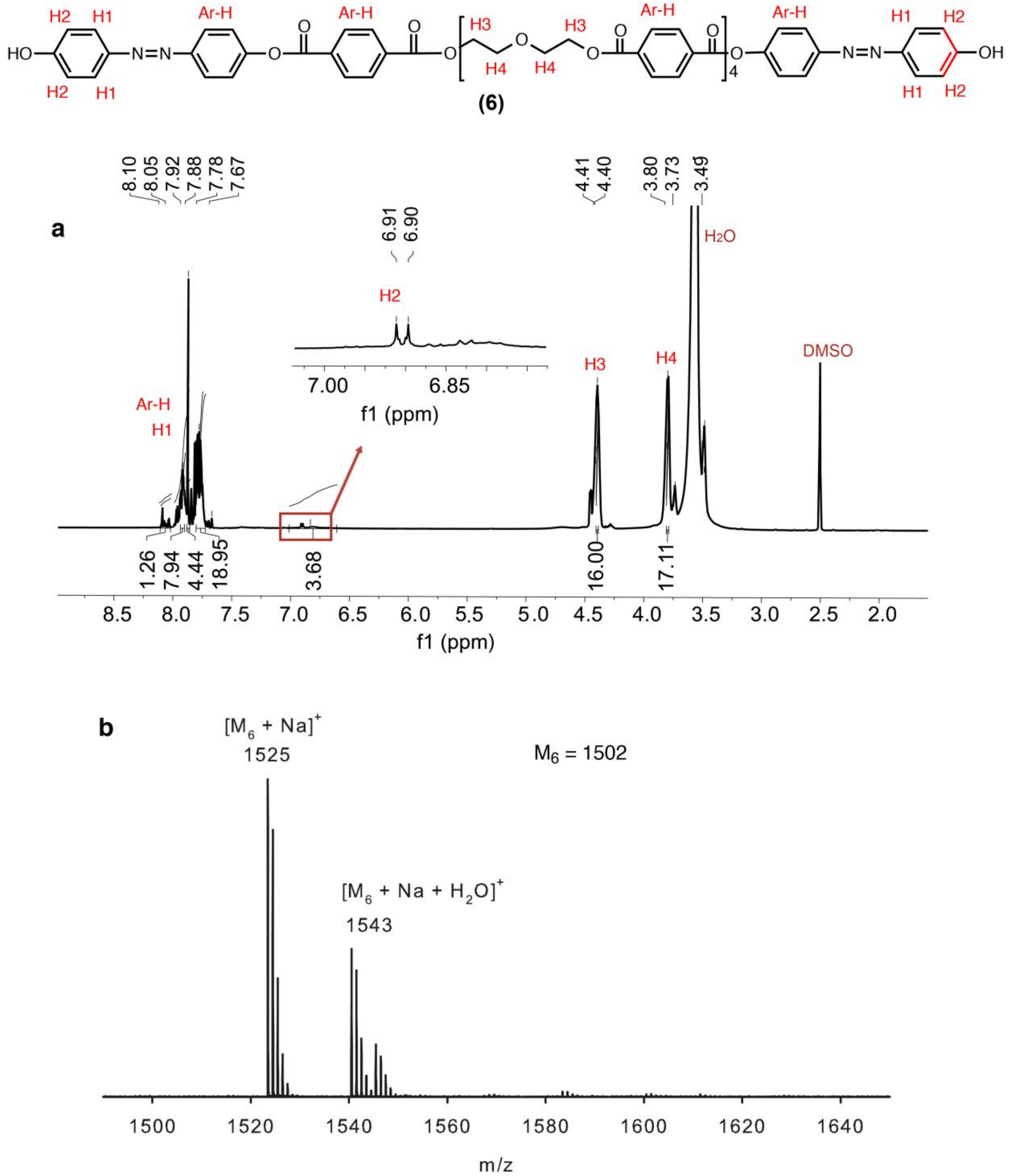

Supplementary Figure 13. Structure and characterization of product 6. ^{1}H NMR spectrum (600 MHz, DMSO–d_6): δ 3.80 (s, 16H, –CH_2–), 4.40 (s, 16H, –CH_2–), 6.90–6.91 (s, 4H, J = 6 Hz, Ar–H), 7.67–7.78 (m, 19H, J = 66 Hz, Ar–H), 7.88 (s, 4H, Ar–H), 7.92–8.10 (m, 9H, Ar–H); ESI–MS (m/z): $[M]^+$ calcd for M_6 ($C_{80}H_{70}N_4O_{26}$), 1502; found, 1525 $[M_6+Na]^+$; 1543 $[M_6+Na+H_2O]^+$.

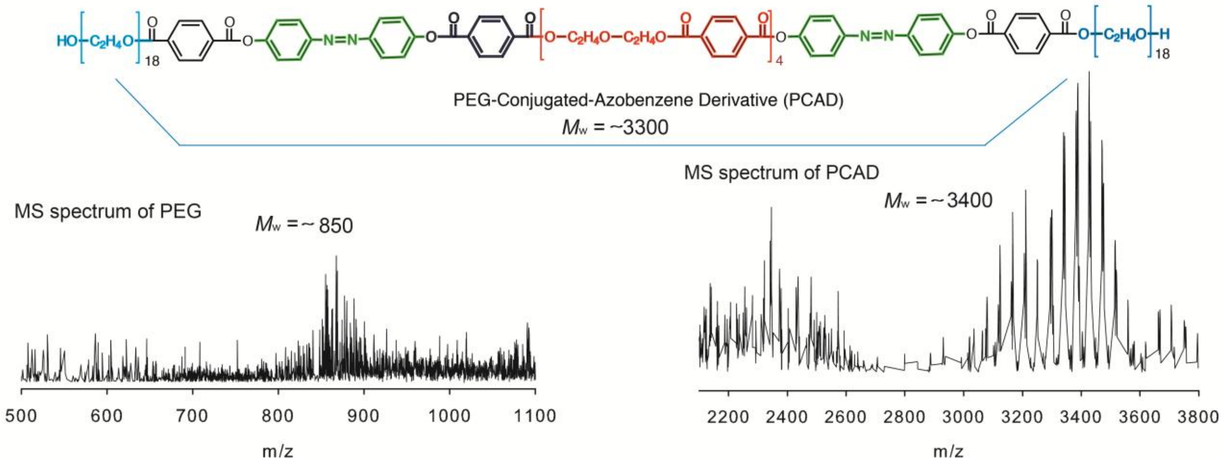

Supplementary Figure 14. Structure and ESI–MS spectrum of PCAD. Melting point: 70–71 °C; UV/Vis: λ_{max} = 365 nm; ESI–MS (*m*/*z*): $[M]^+$ Calcd. for PCAD, ～3300; found, ～3400.

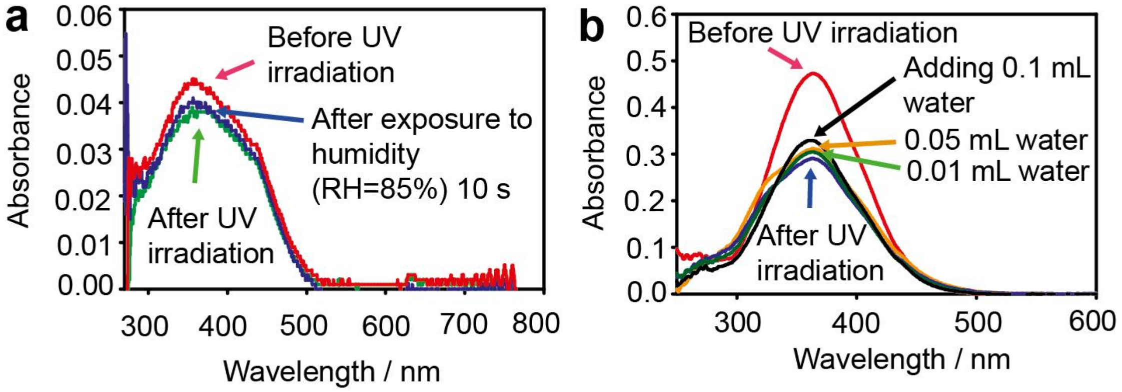

Supplementary Figure 15. Effect of UV light on the UV-Vis spectra of pure PCAD. (a) Spectrum recorded in solid state (pure polyethylene was used as reference). PCAD powder was coated on thin (100 μm) film of polyethylene that is not hygroscopic. Exposure to 85% humidity does not affect the spectrum. (b) Spectrum recorded in dimethylformamide. After the sample was irradiated with UV light, water was added in several portions to the solution to inspect the effect of water on the isomerization at molecular level. The spectra indicate that water does not have a significant effect on the equilibrium.

Supplementary Note 1

In a series of experiments, we established that the motion of the film requires a gradient in humidity rather than a static environment of high humidity. The motion of the film ceases when an equilibrium between the adsorption and desorption of water on both sides is established. The motility can also be suppressed by saturation with water in an environment of extremely high humidity. Variation of the film thickness showed that the optimal thickness for perpetual motion of the film on a moist paper is 35 – 50 μm. Thicker films (>50 μm) had lower turnover frequencies, apparently due to increased stiffness (the turnover frequency was calculated based on continuous monitoring of the sample for one minute. In cases where the motion ceased or the film slid off the surface in less than a minute, the turnover frequency was extrapolated). Thinner films (<35 μm) displayed higher turnover frequencies, however on average they were motile during shorter time due to enhanced adherence to the base. As a typical example, set of PCAD@AG films with thickness of 15 μm were still capable of moving on a moist paper, however the motion was irregular—regardless of whether they were square or rectangular in shape, the films underwent curling, rolling or twisting in an irregular and unpredictable sequence. The time for response was very short (2 s) and wherever a large portion of the surface of the film came in contact to the base, the film readily adhered to it.

Supplementary Note 2

To confirm the changes in hydrogen bonding interactions that occur upon hydration, which are supposedly the direct cause for the humidity-induced mechanical effect of PCAD@AG, we prepared a simple model hybrid material. A low-molecular weight ester which mimics the ester groups in PCAD, methyl 4-nitrobenzoate (MNB), was incorporated in AG. The IR spectra of the film recorded before and after exposure to water vapor and after drying are shown in Supplementary Figure 10. Relative to pure MNB (1719 cm^{-1}), whose structure is devoid of strong hydrogen bonding donors, the carbonyl stretching band in MNB@AG appears as a broad band at 1687 cm^{-1} (the reduced intensity is due to the low concentration of MNB in the matrix). This result indicates hydrogen bonds C=O···HO between MNB and the hydroxyl groups of AG. Upon exposure of the film to humidity, it adsorbed water as it is apparent from the intensification of the water deformation band at 1653 cm^{-1}. The hydration-induced shift of the carbonyl stretching band from 1687 cm^{-1} to 1697 cm^{-1} is in line with depletion of the hydrogen-bonding interactions between MNB and AG as a result of the competitive hydrogen bonding of AG with water. The shift can be reverted by dehydration whereupon hydrogen bonds between the carbonyl groups and AG are re-established.

Supplementary Note 3

By varying the UV light intensity, we were able to control the actuation of PCAD@AG film by slowing down its motion without complete cessation. This photoinduced modulation of the motion can be explained by considering the interplay between the two stimuli from the view of their macroscopic effects (Supplementary Figure 11). It is noteworthy that, as discussed above, the free motion of the film over a moist surface is an exceedingly complex dynamic process that is driven by the interplay between the kinetics of water adsorption/desorption, and the kinematics of the dynamic element as determined by its elastic properties and aspect ratio. Throughout the

process, different parts of the film alternatively come into contact with the moist surface and with air to adsorb and desorb water at different points of time, thereby experiencing continually changing conditions that are difficult to measure and control. Excitation with UV light adds to the complexity of the dynamics of this system with additional strains imposed by the photoisomerization to disturb the kinematic cycle in a process which slows down and eventually ceases the dynamic process.

Supplementary Note 4

As an additional evidence that the hydrogen bonds between agarose and the azobenzene-containing polymer are required for *cis-trans* isomerization, the effect of humidity or water on the UV-Vis spectra of pure PCAD (without agarose) was studied in solid state and in solution (Supplementary Figure 15). The spectra indicate that the *cis-trans* isomerization of azobenzene units in PCAD alone was not affected significantly by the presence of water.

Supplementary Methods

Materials. 4-Aminophenol, sodium nitrite, phenol, urea, sodium carbonate, sodium hydroxide, terephthaloyl chloride, hydrochloric acid (35%), ethanol, chloroform and pyridine were from commercial source (Aldrich) and were used without further purification. Polyethylene glycol (M_r ≈ 850), and diethylene glycol (Aldrich) were dried overnight in vacuum oven at 80 °C before use. Microscopy glass slides (25.4 mm × 76.2 mm × 1.0 mm) from T&Q industries were washed with ethanol and water and dried at 50 °C before casting of the films. All images and videos were recorded with iPhone 4s camera (Apple Inc).

Synthesis of (E)-4,4'-(diazene-1,2-diyl)diphenol (5). The synthetic pathway is shown in Supplementary Figure 1. The precursor **5** was prepared according to a published method[1]. To 4 mL 35% HCl 4-aminophenol (1.0 g, 9.2 mmol) were added. The solution was stirred at room temperature during 30 minutes and then cooled to 0–5 °C with ice. 2 M solution of $NaNO_2$ (4.6 mL) was slowly injected during vigorous stirring. The excess nitrous acid (monitored by potassium iodide-starch paper) was neutralized with urea. Phenol (0.86 g, 9.2 mmol) was dissolved in 2 M NaOH (9.2 mL) and the solution of the diazonium salt was added dropwise to the phenolic solution at 5 °C. The reaction system was kept basic by adding Na_2CO_3 powder to facilitate the formation of diazenes. The reaction mixture was stirred for 2 h at room temperature. The resulting dark red precipitate was filtered off and washed with cold water. Recrystallization from EtOH/H_2O (1:5) gave the desired azobenzene (85%) as dark red solid. ^{1}H NMR (600 MHz; MeOH-*d*4): δ (ppm) = 7.76–7.77 (4H, m), 6.91–6.92 (4H,m); HRMS (m/z): $[M+H]^+$ Calcd. for $C_8H_{10}N_2O_2$, 215.0742; found, 215.0902.

Synthesis and characterization of the PEG-conjugated azobenzene derivative (PCAD). PCAD was prepared in a series of consecutive condensations. The details of the synthetic protocol are described in the Supplementary Figure 1. Briefly, to a 150 mL two-neck flask, 10 mmol diethylene glycol dissolved in 10 mL of chloroform and 10 mol pyridine in large excess were added during vigorous stirring. 10 mmol terephthaloyl chloride in 10 mL chloroform was then

injected at a rate of 0.3 mL minute^{-1} at room temperature under N_2. After the addition, the temperature was increased to 50 ^oC and the mixture was stirred for 5 h. Liquid chromatography–mass spectrometry (LCMS) analysis indicated that the diethylene glycol was consumed completely. Part of the reaction mixture containing the product **3** (around 5 mL) was taken out, evaporated to remove solvent, washed with water and ethanol, dried in vacuum oven overnight and used for analysis (Supplementary Figure 12). To the remaining solution, 1 mmol of terephthaloyl chloride was added to generate the active intermediate (**4**). This step required 2 hours for completion (LCMS). Subsequently, 1 mmol of (*E*)-4,4′-(diazene-1,2-diyl)diphenol (**5**) dissolved in 5 mL chloroform were dropped into the solution, which allows conjugation of photosensitive units to the main chain of the polymer (**6**). After complete transformation of **5** (LCMS), part of the product **6** (around 5 mL) was taken out and the solvent was removed by evaporation. The residue was washed with water, ethanol and chloroform, dried in a vacuum oven at 50 ^oC overnight, and used for characterization (Supplementary Figure 13). 1 mmol terephthaloyl chloride was again added to the remaining reaction mixture to obtain the product **7**. In the last step, 2.3 mmol PEG (2 g, $M_r \approx 850$) were added to obtain the PEG-conjugated azobenzene derivative (PCAD). The solvent was evaporated and the product was poured into 500 mL pure water to remove the pyridinium side product and the excess PEG. The precipitate, collected by suction filtration, was obtained as dark red powder. After triple wash with water/ethanol (50%), the powder was dried in vacuum oven overnight at 50 °C before characterization (Supplementary Figure 14).

Model studies. To study the hydrogen-bonding interaction between the carbonyl and hydroxyl groups in the PCAD@AG film and the changes in the hydrogen bonding network during water adsorption, we used methyl-4-nitrobenzoate (MNB) as a simple model for the ester groups in PCAD. A hybrid MNB@AG film was prepared by following a procedure similar to that used to prepare PCAD@AG. The IR spectrum of dried MNB@AG film was first recorded in ATR mode. The film was then allowed to adsorb water from a moist paper for 5 minutes, and the spectrum was recorded again. As shown in Supplementary Figure 10, in comparison with the IR spectrum of pure MNB, the carbonyl (C=O) band frequency of MNB in agarose exhibited a shift from 1719 cm^{-1} to 1687 cm^{-1} with greatly reduced intensity. This shift indicates formation of hydrogen bonds between C=O and OH in the MNB@AG film. By exposure of the MNB@AG film to humidity, the hydrogen-bonding interaction between C=O of MNB and OH groups of AG was disrupted by water molecules. This resulted in shift of the carbonyl stretching band to 1697 cm^{-1} [2]. Simultaneously, the band 1653 cm^{-1} from the water bending mode was intensified, reflecting the adsorption of water.

Nuclear magnetic resonance (NMR) spectroscopy. The spectra were recorded at 25 °C on Bruker Avance 600 spectrometer at a working frequency of 600 MHz for the ^1H nuclei. All chemical shifts are reported in ppm relative to the signals corresponding to the residual non-deuterated solvents ($CDCl_3$: δ = 7.26 ppm, DMSO–d_6: δ = 2.50 ppm)[3].

Kinematics analysis. The kinematic analysis was carried out by using the software HotShot Link (ver. 1.2) which allows the user to determine (x,y) coordinates of a point with respect to the origin (0,0) on the program window. Inclination of the polymer film tip at different times were extracted as (x,y) by selecting a point on the polymer film tip. In the photoirradiation experiments, the first frame (inclination = 0, time = 0) corresponds to the first frame immediately

after the UV light was switched on. The (x,y) coordinates were extracted at different times. The deflection of the film relative to the position in the first frame was calculated in pixels as $d = [(x_2–x_1)^2 + (y_2–y_1)^2]^{1/2}$, where $x_{1,2}$ and $y_{1,2}$ are the x and y are the coordinates of two consecutive points[4].

Prediction of the maximum load for thin film used as a cantilever crane. The maximal elastic stress of the moist film of PCAD@AG (σ) is around ~35 MPa (Figure 1i in the main text). The cross-sectional area (A) of a film with thickness 200 µm and width 0.5 cm is 200 µm × 0.5 cm. We assume that the maximum load is m. The stress normal to the bending plane ("normal stress") can be expressed as

$$\sigma = F_n / A$$

where σ = 35 MPa, F_n is the normal component force (in N) = mg, and A is the area (in m^2). From $\sigma = mg/A$ we find

$$m = (A \times 35\ \mathrm{MPa})/g = (200\ \mu\mathrm{m} \times 0.5\ \mathrm{cm} \times 35\ \mathrm{MPa})/(9.8\ \mathrm{kg/N}) \approx 3.5\ \mathrm{kg}$$

Supplementary References

1. Chao, M. C., Weng, N. H., Chang, H. C., Jiang, J. C. & Lin, S. H. High–pressure and concentration–dependent studies on C–H–O interactions of binary aqueous mixtures: formic acid/D_2O and acetone/D_2O. *J. Chin. Chem. Soc.* **48**, 603–607 (2001).

2. Blume, A., Hubner, W. & Messnert, G. Fourier Transform Infrared Spectroscopy of 13 C=O-labelled Phospholipids Hydrogen Bonding to Carbonyl Groups. *Biochemistry* **27**, 8240–8249 (1988).

3. Chang, H. C., Jiang, J. C., Chao, M. C., Lin, M. S., Lin, S. H., Chen, H. Y. & Hsueh, H. C. Pressure-dependent studies on hydration of the C–H group in formic acid. *J. Chem. Phys*. **115**, 8032–8037 (2001).

4. Nath, N. K., Pejov, L., Nichols, S. M., Hu, C. H., Saleh, N., Kahr, B. & Naumov, P. Model for photoinduced bending of slender molecular crystals. *J. Am. Chem. Soc*. **136**, 2757−2766 (2014).
